# Astrocytic pyruvate dehydrogenase kinase-2 is involved in hypothalamic inflammation in mouse models of diabetes

**DOI:** 10.1038/s41467-020-19576-1

**Published:** 2020-11-20

**Authors:** Md Habibur Rahman, Anup Bhusal, Jae-Hong Kim, Mithilesh Kumar Jha, Gyun Jee Song, Younghoon Go, Il-Sung Jang, In-Kyu Lee, Kyoungho Suk

**Affiliations:** 1 BK21 Plus KNU Biomedical Convergence Program, Department of Biomedical Science and Department of Pharmacology, School of Medicine, Kyungpook National University, Daegu, Republic of Korea; 2 Department of Neurology, Johns Hopkins University, Baltimore, MD 21205 USA; 3 Department of Medical Science, College of Medicine, Catholic Kwandong University, Gangneung-si, Republic of Korea; 4 Translational Brain Research Center, International St. Mary’s Hospital, Catholic Kwandong University, Incheon, Republic of Korea; 5 Korean Medicine Application Center, Korea Institute of Oriental Medicine, Daegu, 41062 Republic of Korea; 6 Department of Pharmacology, School of Dentistry, Kyungpook National University, Daegu, 700-412 Republic of Korea; 7 Brain Science and Engineering Institute, Kyungpook National University, Daegu, 41944 Republic of Korea; 8 Department of Internal Medicine, School of Medicine, Kyungpook National University Hospital, Daegu, 700-721 Republic of Korea; 9 Research Institute of Aging and Metabolism, Kyungpook National University, Daegu, 700-721 Republic of Korea

**Keywords:** Astrocyte, Molecular neuroscience, Metabolic disorders

## Abstract

Hypothalamic inflammation plays an important role in disrupting feeding behavior and energy homeostasis as well as in the pathogenesis of obesity and diabetes. Here, we show that pyruvate dehydrogenase kinase (PDK)-2 plays a role in hypothalamic inflammation and its sequelae in mouse models of diabetes. Cell type-specific genetic ablation and pharmacological inhibition of PDK2 in hypothalamic astrocytes suggest that hypothalamic astrocytes are involved in the diabetic phenotype. We also show that the PDK2-lactic acid axis plays a regulatory role in the observed metabolic imbalance and hypothalamic inflammation in mouse primary astrocyte and organotypic cultures, through the AMPK signaling pathway and neuropeptidergic circuitry governing feeding behavior. Our findings reveal that PDK2 ablation or inhibition in mouse astrocytes attenuates diabetes-induced hypothalamic inflammation and subsequent alterations in feeding behavior.

## Abstract (web summary)

Hypothalamic inflammation is involved in the pathogenesis of diabetes. The underlying mechanisms are unclear. Here, the authors show that astrocytic PDK2 ablation or inhibition attenuates hypothalamic inflammation in mouse models of diabetes.

## Introduction

Robust and classical inflammatory activation in the hypothalamus has been known to cause illnesses such as anorexia and cachexia^1,2^. Similarly, hypothalamic inflammation is causally important for the pathogenesis of dietary obesity and related metabolic disorders in rodent model^3^ and in human^4,5^. The earlier animal studies revealed that dietary obesity is associated with such hypothalamic inflammation, which is restricted to the mediobasal hypothalamus (MBH) that contains the arcuate nucleus (ARC)^3^. Hypothalamic inflammation has been more recently implicated in a greater range of metabolic disorders that has expanded from obesity to type 2 diabetes^6^, cardiovascular diseases^7^, and metabolic syndrome. Multiple molecular mediators or pathways have been identified as crucial players in hypothalamic inflammation^8^, including nutritional elements^9^, cytokines^10,11^, chemokines^12^, signaling molecules^3^, endoplasmic reticulum and oxidative stress^3,13^, and autophagy dysregulation^14^. Microglia^5,15^, astrocytes^7,16^, perivascular macrophages^17^, and neurons^18^ are the key cell types that contribute to the hypothalamic inflammation associated with metabolic disorders. Emerging evidence suggests a critical role of microglia and astrocytes in hypothalamic dysfunctions associated with obesity and diabetes^19^. Upon dietary insults, microglia, as first-responder cells, play an important role in hypothalamic inflammation and neuronal circuit alterations, promoting obesity and subsequent metabolic complications^5,15,20,21^. Similarly, astrocytes contribute to the alterations in the activity of appetite-regulating neurons in the central hypothalamic melanocortin system and modulate food intake^22^, glucose intolerance, and weight gain in experimental animals with metabolic disorders^7,16^. Inflammatory activation of astrocytes and subsequent structural alterations, changes in the levels of neurotransmitters, and impaired insulin/leptin signaling have been found to be critical molecular causes of such hypothalamic outcomes.

The role of astrocytes in hypothalamic inflammation, however, has mainly been studied in animal models of obesity and type 2 diabetes. Very little is known about the role of hypothalamic astrocytes in type 1 diabetes. Preclinical studies have shown an increased astrocytic activation and proliferation along with increased proinflammatory gene expression in the hypothalamus of rodent models of type 1 diabetes^23,24^. Patients with type 1 diabetes develop polyphagia with sustained hyperglycemia and abnormal body weight^25^, which makes glycemic control more difficult. Aberrant immune responses and a variety of structural and functional anomalies in the hypothalamus are evident in patients with type 1 diabetes^26,27^. Although recent studies acknowledge the role of astrocytes in hypothalamic inflammatory cascades, the contribution of astrocytic intracellular metabolism to hypothalamic inflammation and type 1 diabetes remains unknown. Moreover, most preclinical studies to date have suggested a neuron-centric mechanism. Our proposed role of astrocytes in hypothalamic inflammation and diabetes will pave the way to further mechanistic studies.

Growing evidence suggests that glial (microglial and astrocytic) phenotypic alteration is driven by a shift in the metabolic pathway; especially, switching from mitochondrial oxidative phosphorylation to glycolysis has been found to be associated with inflammatory activation of glia in diverse neuroinflammatory conditions^28–30^. A recent study has highlighted the alteration of mitochondrial metabolism as a major player in microglial activation and hypothalamic inflammation in the diet-induced obesity^31^. With respect to the implication of mitochondrial metabolic alteration in neuroinflammation, we hypothesized that pyruvate dehydrogenase (PDH) kinase (PDK), one of the key regulators of the mitochondrial gatekeeping enzyme PDH complex involved in glucose metabolism^32^, might be a potential target for the study of hypothalamic inflammation in diabetes. PDKs (PDK1-4) are primarily responsible for the regulation of pyruvate dehydrogenase complex activity via phosphorylation of PDH. PDK-mediated phosphorylation and subsequent inactivation of PDH alter glycolytic metabolism and produce lactate as an end product^33^. Our previous studies have identified PDKs as an important regulator of immune cell metabolism and have suggested its widespread expression^34,35^ and critical role in inflammation in the peripheral nervous system. PDKs are also expressed in the central nervous system tissues^36^ and may play a role in impaired glucose metabolism in traumatic brain injury as well as in craniotomy. However, the expression levels of hypothalamic PDKs and their functional role in neurometabolic disorders have not been studied. Although astrocyte metabolic alterations are critical for their inflammatory activation/phenotype^29,30^, the contribution of astrocyte metabolic reprogramming to hypothalamic inflammation and related metabolic disorders, such as diabetes, remains to be explored.

Here, we report that astrocytic PDK2 regulates metabolic and inflammatory pathways that contribute to hypothalamic manifestations of diabetes. We found that diabetes in mice enhances the hypothalamic expression of PDK2 and phosphorylated-PDH (p-PDH), causing a glycolytic metabolic shift along with substantial hypothalamic inflammation. Genetic ablation or hypothalamic inhibition of PDK2 attenuates diabetes-induced neuroinflammation, lactate surge in the hypothalamus, and food intake in mice. In addition, dysregulation of the neuropeptidergic circuitry involved in feeding behavior is improved by deficiency or inhibition of hypothalamic PDK2. Similarly, hypothalamic astrocyte-specific *Pdk2* deficiency reverses the increased food intake caused by diabetes and decreases inflammation as well as the lactate level in the hypothalamus. Mechanistically, studies using primary astrocytes, hypothalamic neuronal cells, and electrophysiological analysis of brain slice cultures demonstrate that PDK2 is a critical regulator of metabolic homeostasis and hypothalamic pathology involved in altered feeding behaviors caused by diabetes.

## Results

### Diabetes enhances the expression of PDK2 and phosphorylated-PDH in mouse hypothalamus

**Fig. 1:**
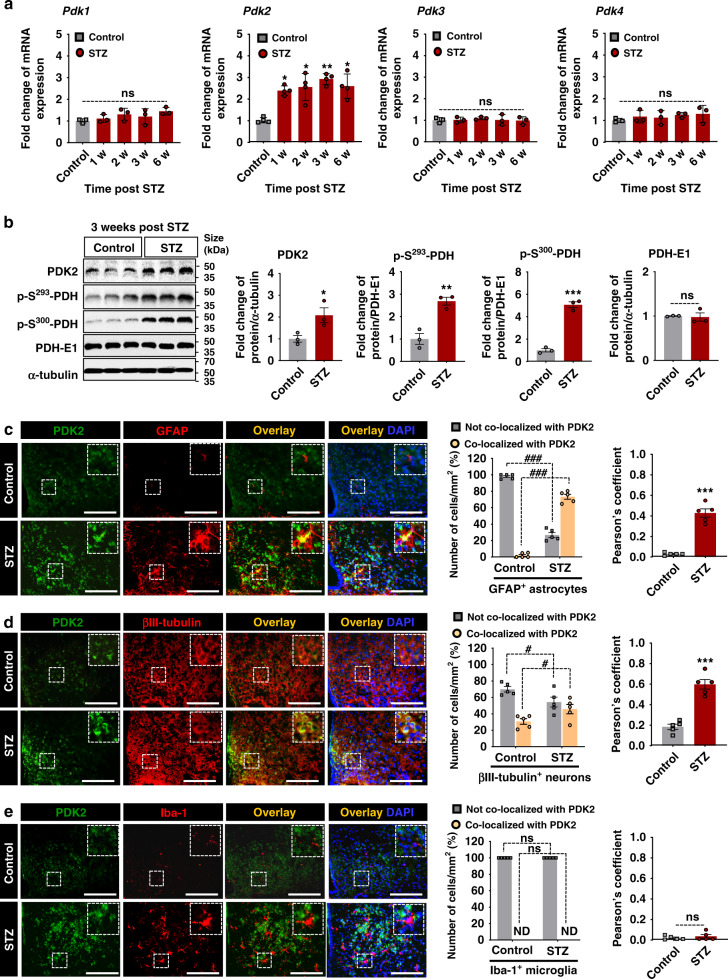
Expression of PDK and phosphorylated-PDH in mouse hypothalamus following diabetes induction. The expression of *Pdk* isoform *(Pdk1-4)* mRNAs in the hypothalamus at 1, 2, 3, and 6 w following STZ injection (**a**) was assessed by real-time RT-PCR. mRNA expression profiles are displayed as the fold increase of gene expression normalized to *Gapdh* (*p = 0.0463, 1 w; *p = 0.0249, 2 w; **p = 0.0063, 3 w; and *p = 0.0212, 6 w for *Pdk2*). PDK2 (*p = 0.0442, STZ), phosphorylated-PDH (p-S^293^-PDH: **p = 0.0048, STZ; and p-S^300^-PDH: ***p = 0.0002, STZ), and PDH-E1 protein levels in the hypothalamus at 3 w post-STZ injection (**b**) were assessed by Western blot analysis. Western blot band quantification for p-PDH was based on normalization to PDH-E1, and PDK2 and PDH-E1 were normalized to α-tubulin. Immunofluorescence analyses show the expression of PDK2 in GFAP-positive astrocytes (**c**), βIII-tubulin-positive neurons (**d**), and Iba-1-positive microglia (**e**) in the hypothalamus at 3 w post-STZ injection. Inserts show the representative double-labeled cells. Quantification of the percentage of PDK2-positive or -negative astrocytes (###*p* = 1.5E-12, not co-localized with PDK2; and ###*p* = 1.6E-12, co-localized with PDK2), neurons (#*p* = 0.0119, not co-localized with PDK2; and #*p* = 0.0143, co-localized with PDK2), or microglia. Pearson’s correlation coefficients for co-localization per mm^2^ (***p = 1.02E-5, STZ for **c**; and ***p = 5.2E-5, STZ for **d**) are shown in the adjacent graphs. Results were obtained from three animals for each condition. Microscopic data were evaluated in five randomly selected fields captured at the same magnification. Scale bars indicate 200 µm. *p < 0.05, **p < 0.01, or ***p < 0.001 versus the vehicle-treated control animals. One-way ANOVA (**a**), two-way ANOVA (**c**–**e**, left) with Tukey’s post hoc test; Student’s *t*-test (**b**–**e**, right), **n** = 3 (**a**, **b**), and *n* = 5 (**c**–**e**); mean ± SEM. Value of “n” indicates the number of animals. Source data are provided as a Source data file. w week(s); STZ streptozotocin; ND not detected; ns not significant.

To understand the PDK-mediated metabolic modulation of hypothalamic inflammation and subsequent pathologies in diabetes, we first examined the expression of PDKs (PDK1–4) in the hypothalamus of mice with type 1 diabetes induced by injecting either a single high dose of streptozotocin (STZ) or multiple low doses (MLDS) using quantitative PCR, Western blot analysis, and immunohistochemical analysis. Enhanced expression of *Pdk2*, but not *Pdk1, Pdk3*, or *Pdk4* mRNA was found in the hypothalamus following a single dose of STZ (Fig. 1a) or MLDS injection (Supplementary Fig. 1a). Similarly, PDK2 and p-PDH protein (p-S^293^-PDH and p-S^300^-PDH) levels were markedly increased in the hypothalamus at 3 weeks post-STZ (Fig. 1b) and MLDS (Supplementary Fig. 1b) injection. Next, the immunohistochemical analysis revealed that PDK2 is predominantly expressed in the arcuate nucleus (ARC) at high levels but not in other areas of diabetic mouse brain (Supplementary Fig. 1c). In the ARC, PDK2 is expressed primarily in GFAP-positive astrocytes (Fig. 1c) and βIII-tubulin-positive neurons (Fig. 1d) but not in Iba-1-positive microglia (Fig. 1e). In addition, we also used a mouse model of obesity and type 2 diabetes induced by high-fat diet (HFD) feeding. HFD-fed mice showed substantially higher levels of PDK2 and p-PDH (Supplementary Fig. 6a, b) in the hypothalamus, and GFAP-positive astrocytes and βIII-tubulin-positive neurons were the major cell types expressing PDK2 (Supplementary Fig. 6c–e). To investigate whether PDK2 is expressed in neurons differentially, we performed immunostaining of PDK2 and AgRP or POMC in serial brain sections isolated from diabetic mice, which revealed that PDK2 is predominantly expressed in AgRP-positive neurons (Supplementary Fig. 1d) but not in POMC-positive neurons (Supplementary Fig. 1e) at 3 weeks post-STZ injection. These findings led us to further investigate the role of PDK2 in hypothalamic pathology associated with diabetes.

To confirm whether PDK2 is a major regulator of PDH phosphorylation in the diabetic hypothalamus, we measured the protein levels of PDKs and p-PDH in hypothalamus isolated from WT and *Pdk2* KO mice after 3 weeks of STZ injection. Consistent with the expression levels of *Pdks* mRNAs, enhanced expression of PDK2 protein, but not PDK1, 3, and 4 proteins, was observed in the diabetic hypothalamus. Furthermore, *Pdk2* deficiency significantly attenuated the diabetes-induced increase in p-S^293^-PDH and p-S^300^-PDH protein levels (Supplementary Fig. 1f). These findings suggest that PDK2 is a major regulator of PDH phosphorylation in the hypothalamus, which may play an important role in diabetes.

### *Pdk2* deficiency attenuates diabetes-induced hypothalamic inflammation, lactate surge, increased food intake, and hyperglycemia in mice

**Fig. 2:**
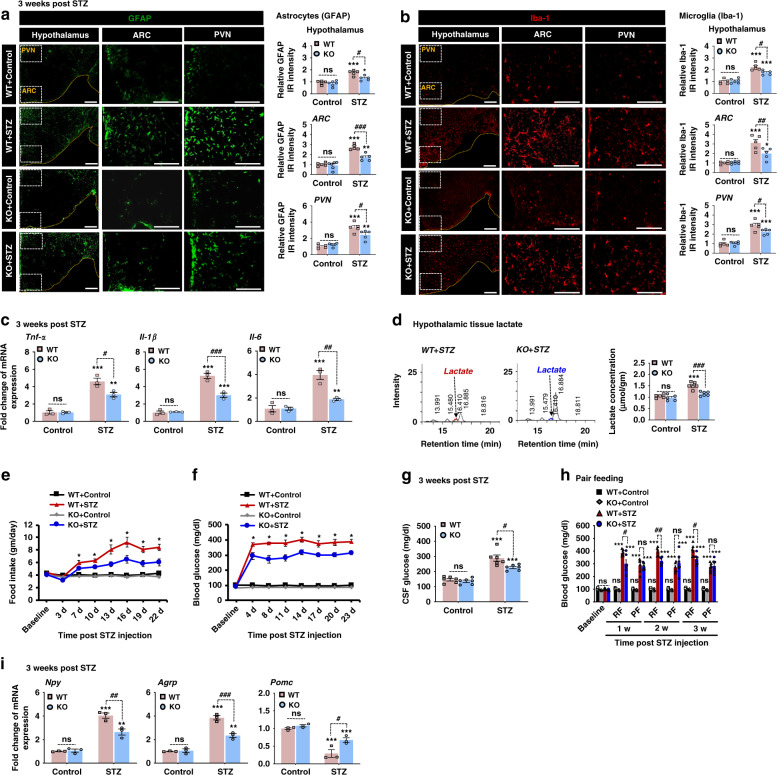
*Pdk2*-deficiency attenuates the diabetes-induced increase in proinflammatory cytokines, gliosis, lactate level in the hypothalamus, food intake, and blood and CSF glucose levels in mice. GFAP (#*p* = 0.0101, KO + STZ for hypothalamus; ###p = 0.0003, KO + STZ for ARC; and #*p* = 0.0192, KO + STZ for PVN) and Iba-1 (#*p* = 0.0170, KO + STZ for hypothalamus, ##*p* = 0.0090, KO + STZ for ARC; and #*p* = 0.0277, KO + STZ for PVN) immunofluorescence staining and image analysis were performed using WT and *Pdk2*-deficient mice at 3 w post-STZ injection (**a**, **b**). Microscope data were gathered using five to six randomly selected fields captured at the same magnification. Scale bars indicate 400 µm (**a**, **b**, hypothalamus) and 200 µm (**a**, **b**, ARC and PVN). The relative expression of *Tnf-α* (#p = 0.0112, KO + STZ), *Il-1β* (###p = 0.0003, KO + STZ), and *Il-6* (##p = 0.0016, KO + STZ) mRNAs in the hypothalamus after 3 w of STZ injection (**c**) was evaluated by real-time RT-PCR. Results for mRNA expression are displayed as the fold increase in gene expression normalized to *Gapdh*. Lactate concentration in hypothalamic tissues collected from WT and *Pdk2* KO mice at 3 w post-STZ/vehicle injection was measured with an HPLC analyzer (**d**). The quantification of lactate concentration (###*p* = 0.0002, KO + STZ) from HPLC graphs is shown. Food intake (*p = 0.0017, KO + STZ at 22 d) (**e**) and fasting blood (*p = 0.0033, KO + STZ at 23 d) (**f**) and CSF glucose (#*p* = 0.0101, KO + STZ) (**g**) levels in mice were assessed following STZ injection at the indicated time points. Fasting blood glucose levels were measured in the pair-feeding condition following STZ administration (**h**). The pair-feeding condition was established based on the consumption of food by diabetic KO animals (*p* = 0.9997, KO + STZ for PF at 3 w). The relative expression of *Npy* (##*p* = 0.0031, KO + STZ), *Agrp* (###*p* = 0.0004, KO + STZ), and *Pomc* (#*p* = 0.0158, KO + STZ) mRNA in the hypothalamus after 3 w of STZ injection (**i**) was evaluated by real-time RT-PCR. mRNA expression results are displayed as the fold increase of gene expression normalized to *Gapdh*. *p < 0.05, **p < 0.01, or ***p < 0.001 versus the WT/KO control animals (**a**–**d**, **g**–**i**) or STZ-injected animals (**e**, **f**); #*p* < 0.05 or ##*p* < 0.01 versus indicated groups. Two-way ANOVA with Tukey’s post hoc test, *n* = 6 for KO + Control and 5 for other groups (**a**, **b**), *n* = 5 for control and 6 for STZ groups (**d**), *n* = 6 (**e**–**h**), and *n* = 3 (**c**, **i**); mean ± SEM. Value of “*n*” indicates the number of animals. Source data are provided as a Source data file. w week(s); d day(s); WT wild-type; KO knock out; STZ streptozotocin; ARC arcuate nucleus; PVN paraventricular nucleus; IR immunoreactivity; RF regular feeding; PF pair feeding; ns not significant.

To evaluate whether PDK2 is involved in diabetes-induced hypothalamic inflammatory processes and subsequent metabolic and behavioral dysfunction, we first performed immunofluorescence analyses that showed increased immunoreactivity for GFAP (Fig. 2a) and Iba-1 (Fig. 2b) in the hypothalamus at 3 weeks after STZ injection. This indicated a heightened level of astrocytic and microglial activation and proliferation under such conditions. We also observed a hypertrophic morphology with thick processes. Importantly, such gliosis has been observed predominantly in the ARC and paraventricular nucleus (PVN) regions of the hypothalamus of STZ-induced diabetic mice (Fig. 2a, b) but is reduced in *Pdk2*-deficient mice. On the other hand, the diabetes-induced expression of proinflammatory cytokines such as *Tnf-α*, *Il-1β*, and *Il-6* was substantially diminished by *Pdk2*-deficiency (Fig. 2c). We also examined the role of PDK2 in HFD-induced hypothalamic inflammation using *Pdk2* KO mice. Similarly, *Pdk2*-deficient HFD-fed mice showed lower levels of inflammatory cytokines (Supplementary Fig. 7a) and gliosis (Supplementary Fig. 7b) in the hypothalamus after 16 weeks of HFD feeding. These findings imply that PDK2 has an important role in diabetes-associated hypothalamic inflammation.

The increased expression level of PDK2 in the diabetic hypothalamus led us to assess the production of lactate in that tissue. As expected, a higher level of lactate was observed in the hypothalamus of diabetic mice (Fig. 2d). However, *Pdk2*-deficiency reduced this lactate surge in the hypothalamus of diabetic mice. These results are consistent with the up-regulation of PDK2 in the diabetic hypothalamus (Fig. 1). Thus, increased PDK2 inhibits PDH activity, which causes a glycolytic shift accompanying the diversion of pyruvate to increased lactate production.

Next, we compared the feeding behavior (Fig. 2e), glycemic state (Fig. 2f, g), and body weight (Supplementary Fig. 2a) changes of diabetic-WT mice with diabetic*-Pdk2* KO mice. The diabetes-induced increased food intake (Fig. 2e), as well as the blood (Fig. 2f) and CSF (Fig. 2g) glucose levels, were substantially reduced by *Pdk2*-deficiency following STZ injection. To determine whether the observed lower blood glucose level was caused by changes in food intake shown by *Pdk2* KO mice, we performed a pair-feeding study in which the same amount of food consumed by diabetic *Pdk2* KO mice was given to WT-diabetic mice and to control animal groups (Fig. 2h). We found that restricting the amount of food given to WT-diabetic mice reduces blood glucose to levels found in *Pdk2-*deficient mice following diabetes. These results suggest that the lower blood glucose levels observed in *Pdk2* KO mice are caused by reduced food intake following diabetes. However, diabetic mice of both genotypes had lower body weight when compared with vehicle-injected control animals (Supplementary Fig. 2a).

Furthermore, we measured the expression levels of metabolically relevant food intake-regulating neuropeptide (i.e., orexigenic neuropeptide Y, *Npy*/agouti-related peptide, *AgRP*, and anorexigenic proopiomelanocortin, *Pomc*) mRNA in the hypothalamus. Interestingly, diabetic mice had higher expression levels of *Npy* and *Agrp* and lower levels of *Pomc* mRNA at 3 weeks post-STZ injection (Fig. 2i). However, *Pdk2* deficiency decreased the expression levels of *Npy* and *Agrp* and increased the expression of *Pomc* mRNA. Insulin and leptin are known to regulate feeding behavior by modulating hypothalamic NPY, AgRP, and POMC neuropeptides^37^. To determine whether the reduced food intake shown by *Pdk2*-deficient mice following diabetes is due to changes in systemic insulin or leptin levels, we collected plasma from WT and *Pdk2* KO mice at 3 weeks post-STZ injection. Systemic insulin and leptin levels were not significantly different between WT and *Pdk2* KO mice, although they were lower in the samples collected from both WT and *Pdk2* KO diabetic mice when compared to vehicle-injected control animals (Supplementary Fig. 2b). We also compared the level of ketone body (β-hydroxybutyrate) in the plasma collected from WT and *Pdk2* KO mice at 3 weeks post-STZ injection. Ketone bodies are known to serve as an alternative energy source of glucose during malnutrition and are involved in the regulation of food intake by modulation of brain energy metabolism and neuropeptide circuitry^38^. Although the plasma level of ketone body was increased following STZ-induced diabetes, it was not significantly different between WT and *Pdk2* KO mice (Supplementary Fig. 2c).

We also used an HFD-induced obesity and type 2 diabetes mouse model to assess calorie intake (Supplementary Fig. 8a), blood glucose level (Supplementary Fig. 8b), body size (Supplementary Fig. 8c), cold-induced thermogenesis capacity (Supplementary Fig. 8d), and food intake-related neuropeptide expression (Supplementary Fig. 8e) in WT and *Pdk2* KO mice. *Pdk2*-deficient mice showed reductions in calorie intake, fasting blood glucose, and body size when compared with WT animals following HFD feeding. Similarly, *Pdk2* deficiency protected mice from HFD-induced thermogenesis impairment after 16 weeks of HFD feeding, suggesting that PDK2 is an important regulator of energy metabolism. Taken together, it appears that PDK2 plays a crucial role in the hypothalamic pathogenesis of altered feeding behavior and subsequent alteration in energy metabolism associated with both type 1 and 2 diabetes.

### Inhibition of hypothalamic inflammation attenuates increased food intake caused by diabetes

It has been reported that STZ-induced type 1 diabetes increases glial activation and proliferation along with proinflammatory gene expression in the mouse hypothalamus^23,24^. Our data (Fig. 2a–c) and data from previous reports suggest that inflammation is conspicuous in the hypothalamus of type 1 diabetic mice. However, the role of hypothalamic inflammation and its pathological consequences involved in type 1 diabetes-induced alterations in feeding behavior have not been resolved.

**Fig. 3:**
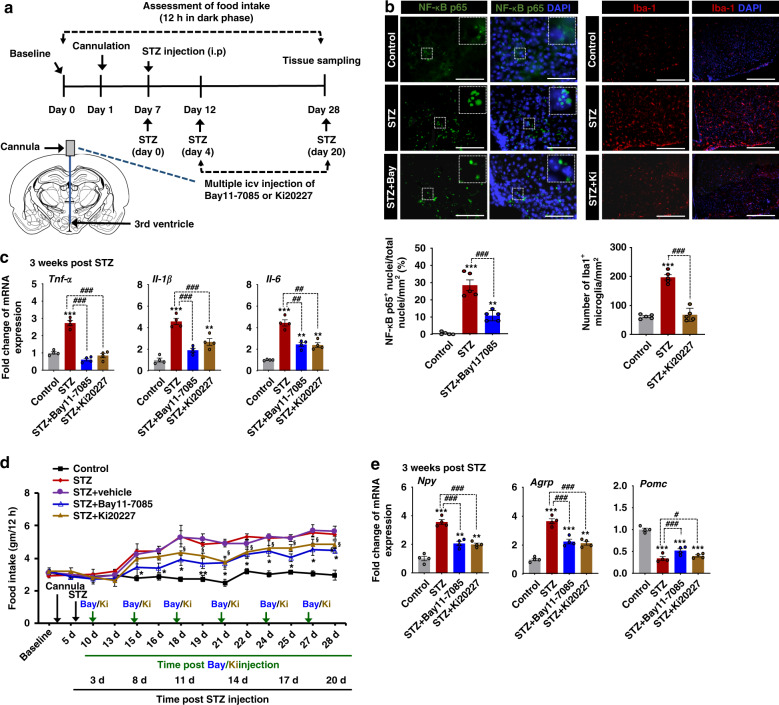
Effects of pharmacological inhibition of hypothalamic inflammation on feeding behavior and expression of food intake-regulating neuropeptides in the diabetic mouse hypothalamus. Bay 11-7085 (an inhibitor of I-κB phosphorylation, 1 µg) and Ki20227 (an inhibitor of CSFR1, 1 µg) solution were administered via multiple icv injections starting from 4 d post-STZ injection to the following indicated time points. The schematic diagram represents the timeline of experimentation and the route of administration of inhibitors (**a**). Immunofluorescence analyses show the immunoreactivity of NF-κB p65 and Iba-1 (**b**) in the hypothalamus at 3 w post-vehicle/STZ injection. Inserts show the translocation of NF-κB p65 in DAPI-positive nuclei. Quantification of the percentage of NF-κB p65-positive nuclei (###*p* = 9.3E-5, STZ + Bay11-7085) and the total number of Iba-1-positive microglia (###*p* = 2.5E-7, STZ + Ki20227) per mm^2^ are shown in the adjacent images. Microscope data were gathered using six randomly selected fields captured at the same magnification. Scale bars indicate 100 µm (left) and 200 µm (right). The expression of proinflammatory cytokines such as *Tnf-α* (###*p* = 6.7E-8, STZ + Bay11-7085; and ###*p* = 2.5E-7, STZ + Ki20227), *Il-1β* (###*p* = 3.7E-5, STZ + Bay11-7085; and ###*p* = 0.0008, STZ + Ki20227), and *Il-6* (#*p* = 0.0120, STZ + Bay11-7085; and ##*p* = 0.0010, STZ + Ki20227) was assessed by real-time RT-PCR (**c**). Food intake (*p = 0.0160, STZ + Bay11-7085; and ^§^*p* = 0.0323, STZ + Ki20227 at 28 d) was measured (**d**), and the expression of *Npy* (###*p* = 0.0006, STZ + Bay11-7085; and ###*p* = 0.0002, STZ + Ki20227), *Agrp* (###*p* = 0.0003, STZ + Bay11-7085; and ###*p* = 0.0004, STZ + Ki20227), and *Pomc* (###*p* = 0.0001, STZ + Bay11-7085; and #*p* = 0.0110, STZ + Ki20227) mRNA was assessed by real-time RT-PCR (**e**). Results for mRNA expression are displayed as the fold increase of gene expression normalized to *Gapdh*. * or ^§^*p* < 0.05 or **p < 0.01 or ***p < 0.001 versus WT/KO control animals (**b**, **c**, **e**) or STZ-injected animals (**d**). One-way ANOVA (**b**, **c**, **e**) and two-way ANOVA with Tukey’s post hoc test (**d**), *n* = 5 (b), *n* = 6 (**d**), and *n* = 4 (**c**, **e**); mean ± SEM. Value of “*n*” indicates the number of animals. Source data are provided as a Source data file. d day(s); STZ streptozotocin; Bay Bay 11-7085; Ki Ki20227.

A crucial contribution of diabetes-induced hypothalamic inflammation to altered feeding behaviors was ascertained via pharmacological inhibition of NF-κB activation and depletion of microglia in the hypothalamus following STZ injection. Bay 11-7085, an inhibitor of I-κB phosphorylation, was used to inhibit NF-κB activation, and Ki20227, an inhibitor of colony stimulating factor 1 receptor (CSF1R), was used to reduce microglial populations in the diabetic hypothalamus. To determine whether intracerebroventricular (icv) administration of Bay 11-7085 and Ki20227 inhibits diabetes-induced hypothalamic inflammation, we measured the number of NF-κB p65-positive nuclei and Iba1-positive microglia, respectively (Fig. 3b), and the mRNA levels of inflammatory cytokines such as *Tnf-α*, *Il-1β*, and *Il-6* (Fig. 3c) at the end of the behavioral studies. Significantly lower levels of cytokines, fewer cells showing NF-κB p65 nucleus translocation, and Iba-1 immunoreactivity in the hypothalamus were observed in Bay 11-7085- and Ki20227-treated diabetic mice. Multiple icv injections of Bay 11-7085 (1 µg) and Ki20227 (1 µg) (Fig. 3a) significantly attenuated diabetes-induced increase in food intake (Fig. 3d). In addition, pharmacological inhibition of hypothalamic inflammation reduced diabetes-induced *Npy* and *Agrp* mRNA expression and increased *Pomc* mRNA levels when compared with the animals injected with STZ alone (Fig. 3e). These findings suggest that hypothalamic inflammation is the key to altered feeding behaviors and related pathologies caused by diabetes.

### *Pdk2* deficiency attenuates the high glucose-induced inflammatory activation of astrocytes and their glycolytic shift

**Fig. 4:**
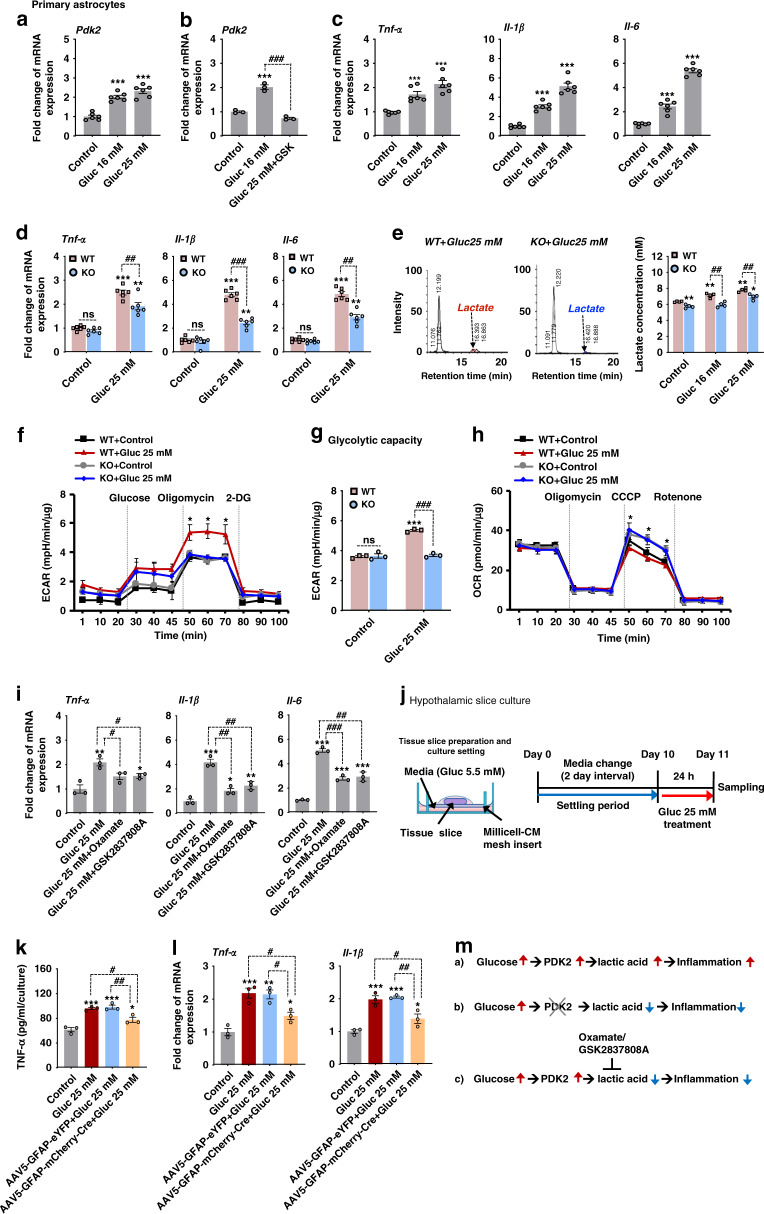
*Pdk2*-deficiency attenuates high glucose-induced inflammatory activation and altered glycolytic metabolism in astrocytes. The relative expression of *Pdk2* (***p = 7.6E-6, Gluc 16 mM; and ***p = 3.6E-7, Gluc 25 mM) (**a**), *Tnf-α* (***p = 0.0001, Gluc 16 mM; and ***p = 8.6E-6 Gluc 25 mM), *Il-1β* (***p = 2.2e-5, Gluc 16 mM; and ***p = 2.7E-9, Gluc 25 mM), and *Il-6* (***p = 1.8E-5, Gluc 16 mM; and ***p = 7.5E-12, Gluc 25 mM) mRNA in cultured astrocytes was assessed after high glucose (16 mM and 25 mM) treatment for 24 h (**c**). Similarly, the expression of *Pdk*2 mRNA (###*p* = 1.7E-5, Gluc 25 mM + GSK) in astrocytes was assessed after co-treatment with high glucose (25 mM) and GSK0660 (a PPARβ/δ antagonist, 1 µM) for 24 h (**b**). The expression of *Tnf-α* (##*p* = 0.0022, KO + Gluc 25 mM), *Il-1β* (###*p* = 2.3E-9, KO + Gluc 25 mM) and *Il-6* (###*p* = 0.0002, KO + Gluc 25 mM) mRNA was examined in cultured astrocytes isolated from WT and *Pdk2* KO mice (**d**). Extracellular lactate was assessed after primary astrocytes isolated from WT and *Pdk2* KO mice were exposed to high glucose for 24 hr by HPLC analysis (**e**). ECAR (*p = 0.0331, KO + Gluc 25 mM at 60 min; and ###p = 7.2E-6, KO + Gluc 25 mM at glycolytic capacity) (**f**–**g**) and OCR (*p = 0.0289, KO + Gluc 25 mM at 60 min) (**h**) were measured after primary astrocytes isolated from WT and *Pdk2* KO mice were treated with high glucose (25 mM) for 24 h by an XF 24 Seahorse analyzer. Relative expression of *Tnf-α* (#*p* = 0.0340, Gluc 25 mM+Oxamate; and #*p* = 0.0246, Gluc 25 mM+GSK2837808A), *Il-1β* (##*p* = 0.0080, Gluc 25 mM + Oxamate; and ##p = 0.0020, Gluc 25 mM + GSK2837808A)*, Il-6* (###p = 2.1E-5, Gluc 25 mM+Oxamate; and ##*p* = 0.0012, Gluc 25 mM + GSK2837808A) mRNA in astrocytes was assessed after co-treatment with high glucose (25 mM) and Oxamate (5 mM) or GSK2837808A (2 µM) for 24 h (**i**). The schematic diagram represents the timeline and condition for the hypothalamic slice culture experiment (**j**). Levels of TNF-α protein (##*p* = 0.0099, AAV5-GFAP-mCherry-Cre + Gluc 25 mM) in the culture media (**k**) and expression of *Tnf-α* (#p = 0.0294, AAV5-GFAP-mCherry-Cre+Gluc 25 mM) and *Il-1β* (##*p* = 0.0058, AAV5-GFAP-mCherry-Cre + Gluc 25 mM) mRNAs (**l**) in the cultured hypothalamus isolated from AAV5-GFAP-eYFP or AAV5-GFAP-mCherry-Cre-injected mice were assessed after high glucose treatment. Protein level was measured by ELISA. mRNA expression was assessed by real-time RT-PCR, and results are displayed as the fold increase of gene expression normalized to *Gapdh*. *p < 0.05, **p < 0.01, or ***p < 0.001 versus the non-treated group; #*p* < 0.05, ##*p* < 0.01, or ###*p* < 0.001 versus indicated groups. One-way ANOVA (**a**–**c**, **i**, **k**, **l**) and two-way ANOVA with Tukey’s post hoc test (**d**–**h**), *n* = 6 (**a**, **c**, **d**), *n* = 3 (**b**, **i**, **k**, **l**), and *n* = 3–4 (**e**–**h**); mean ± SEM. Value of “*n*” indicates the number of sister wells in culture plates. Source data are provided as a Source data file. A diagram showing the hypothetical involvement of the PDK2-lactic acid axis in high glucose-induced inflammatory activation of astrocytes (**m**). Gluc glucose; GSK GSK0660.

To investigate the mechanistic relationships among high glucose, PDK2, lactic acid, and inflammation using primary astrocyte and hypothalamic slice culture models, we first examined the effect of high glucose (16 or 25 mM) on PDK expression. Exposure of cultured astrocytes to high glucose for 24 h substantially enhanced the expression of *Pdk2* (Fig. 4a) but not *Pdk1*, *Pdk3*, or *Pdk4* mRNA (Supplementary Fig. 2d) when compared to expression levels in control astrocytes maintained in low glucose (5.5 mM). It has been reported that the peroxisome proliferator-activated receptor (PPAR)-β/δ is a transcription factor that regulates *Pdk2* gene expression^39^. To determine whether it is involved in high glucose-induced expression of *Pdk2* mRNA in astrocytes, we first confirmed the expression of *Pparβ/δ* mRNA in primary astrocytes following high glucose treatment (25 mM) (Supplementary Fig. 2e). However, the glucose treatment did not alter the expression level of *Pparβ/δ* mRNA. Next, we co-treated primary astrocytes with high glucose and a small-molecule inhibitor of PPARβ/δ (GSK0660, 1 µM). The resulting data revealed that GSK0660 exposure inhibits high glucose-induced expression of *Pdk2* mRNA in astrocytes (Fig. 4b), suggesting that PPARβ/δ mediates the glucose-induced *Pdk2* expression.

To study whether hyperglycemia can cause inflammatory activation of astrocytes and to assess the role of PDK2 in this condition, we compared glucose responses of astrocytes isolated from WT and *Pdk2* KO early postnatal mouse brain tissues. Following 24 hr exposure to high glucose (16 or 25 mM), cultured WT astrocytes showed enhanced expression of *Tnf-α, Il-1β*, and *Il-6* mRNAs (Fig. 4c). This enhanced cytokine expression was reduced in *Pdk2*-deficient astrocytes (Fig. 4d). Similarly, we also treated primary astrocytes with palmitate, a fatty acid, and one of the key factors implicated in HFD-induced hypothalamic inflammation. Exposure of cultured astrocytes to palmitate (200 µM) for 48 h significantly increased the expression of *Pdk2* (Supplementary Fig. 7c), *Tnf-α*, *Il-1β, and Il-6* mRNAs (Supplementary Fig. 7d). However, lower expression levels of these cytokines were observed in *Pdk2* KO astrocytes. These findings indicate that PDK2 is a key player in high glucose/palmitate-induced inflammatory activation of astrocytes and expression of inflammatory cytokines.

To determine the effect of high glucose-induced expression of *Pdk2* on glycolytic metabolism in astrocytes and lactate surge, we first measured extracellular lactate levels in primary astrocytes treated with high glucose (16 or 25 mM) for 24 hr. HPLC analysis revealed that exposure of WT astrocytes to high glucose increased the extracellular lactate level, which was lower in high glucose-treated *Pdk2*-deficient astrocytes (Fig. 4e). Next, we assessed astrocytic glycolysis and extracellular acidification rate (ECAR) under high- glycemic conditions by using a Seahorse XF-24 extracellular flux analyzer. High glucose treatment of cultured astrocytes for 24 h markedly increased ECAR (Fig. 4f) via enhancement of astrocytic glycolytic capacity (Fig. 4g), and the level of ECAR was found to be lower in *Pdk2* KO astrocytes. In addition, *Pdk2* KO astrocytes showed a higher oxygen consumption rate (OCR) than WT astrocytes following high glucose treatment (Fig. 4h). These findings suggest that hyperglycemia promotes astrocytic glycolysis through PDK2 up-regulation; this process results in increased lactic acid production, creating an acidic extracellular microenvironment that may favor further enhanced inflammatory activation of glial cells and the release of proinflammatory cytokines. To test the effect of hyperglycemia-induced production of lactic acid on the enhanced expression of inflammatory cytokines in astrocytes, we co-treated astrocytes with high glucose (25 mM) followed by lactate dehydrogenase (LDH) inhibitors (oxamate/GSK2837808A). The resulting data revealed that inhibiting astrocyte lactic acid production attenuates high glucose-induced expression of *Tnf-α, Il-1β*, and *Il-6* mRNA (Fig. 4i).

To mimic astrocytic confrontation with high glucose in situ, we used organotypic hypothalamus slice cultures that characterize highly preserved cytoarchitectures and complex neuronal network functions (Fig. 4j). Exposure of the slice cultures to high glucose (25 mM) for 24 h significantly increased the levels of TNF-α protein in culture media (Fig. 4k) and *Tnf-α, Il-1β* mRNA in tissues (Fig. 4l). However, selective deletion of astrocytic PDK2 from hypothalamus (generated by bilateral injection of AAV5-GFAP-mCherry-Cre into *Pdk2* floxed mice 2 weeks before culture) attenuated the levels of high glucose-induced inflammatory cytokines.

Furthermore, PDK2-expressing neurons may also contribute to a lactate surge in the diabetic hypothalamus; this is supported by our in vitro study showing that mHypoE-N41 (embryonic mouse hypothalamic cells expressing NPY and AgRP) cells exposed to high glucose release higher levels of lactate when compared to low-glucose-treated controls (Supplementary Fig. 2f). Taken together, these results suggest that the PDK2-lactic acid axis might be a key component of hypothalamic inflammation (Fig. 4m) and subsequent pathologies associated with diabetes.

### Pharmacological inhibition of hypothalamic PDK2 and subsequent lactate production diminishes diabetes-induced local inflammation and food intake

**Fig. 5:**
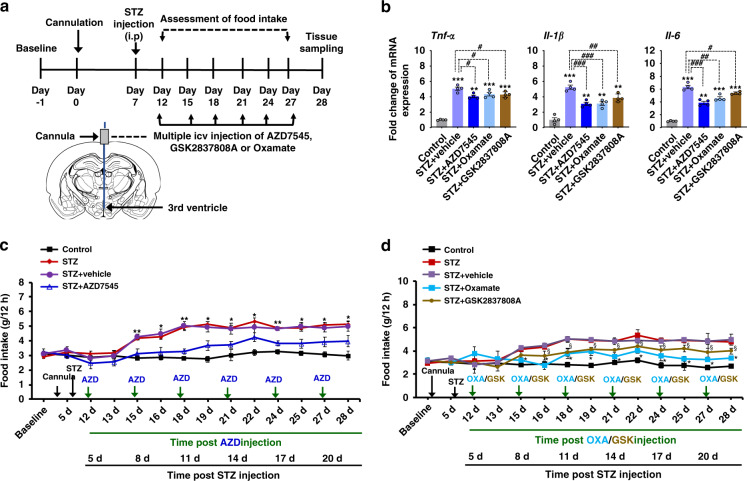
Pharmacological inhibition of PDK2 and lactate production in the mouse hypothalamus attenuates diabetes-induced inflammatory cytokines and food intake. To determine the role of hypothalamic PDK2 or lactic acid in the regulation of food intake, we administered AZD7545 (6.4 nM of CSF concentration), GSK2837808A (2 μM of CSF concentration), or oxamate (25 μg) by multiple icv injections into mice starting from 5 d post-STZ injection to the following indicated time points. The schematic diagram represents the timeline of experimentation and route of administration of the inhibitors (**a**). The relative expression of *Tnf-α*, *Il-1β*, and *Il-6* mRNA in the hypothalamic tissues isolated from AZD7545 or GSK2837808A or oxamate and STZ-injected mice was evaluated by real-time RT-PCR (**b**). Food intake (**c**, **d**) was assessed following AZD7545, GSK2837808A, or oxamate and STZ administration. Results for mRNA expression are displayed as the fold increase of mRNA expression normalized to *Gapdh*. *p or ^§^*p* < 0.05, **p < 0.01, or ***p < 0.001 versus the vehicle-treated control animals (**b**) or STZ + vehicle-treated animals (**c**, **d**); #*p* < 0.05, ##*p* < 0.01, or ###*p* < 0.001 versus indicated groups. One-way ANOVA (**b**) and two-way ANOVA with Tukey’s post hoc test (**c**, **d**), *n* = 4 (**b**), and *n* = 9 for STZ and 5 for other groups (**c**, **d**); mean ± SEM. Value of ‘n’ indicates the number of animals. Source data are provided as a Source data file. d day(s); STZ streptozotocin.

The crucial contribution that hypothalamic PDK2 and lactic acid production make to the pathogenesis of diabetes-induced hypothalamic inflammation and altered feeding behavior in mice was ascertained by pharmacological inhibition of hypothalamic PDK2 and LDH. AZD7545 was used to inhibit PDK2, and the LDH inhibitors GSK2837808A or oxamate were used to suppress lactic acid production (Fig. 5a). Multiple icv injections of AZD7545 (6.4 nM of CSF concentration), GSK2837808A (2 µM of CSF concentration) or oxamate (25 µg) significantly attenuated the diabetes-induced expression of proinflammatory cytokines *Tnf-α, Il-1β*, and *Il-6* mRNAs, in the hypothalamus (Fig. 5b). In addition, pharmacological inhibition of hypothalamic PDK2 (Fig. 5c) and lactic acid production (Fig. 5d) diminished increased food intake. These findings suggest that the hypothalamic PDK2-lactic acid axis plays a crucial role in local neuroinflammation and subsequent dysregulation of neuropeptide circuitry involved in feeding behavior in diabetes.

### PDK2 overexpression in the hypothalamus reverses the effects of *Pdk2* deficiency in diabetes

**Fig. 6:**
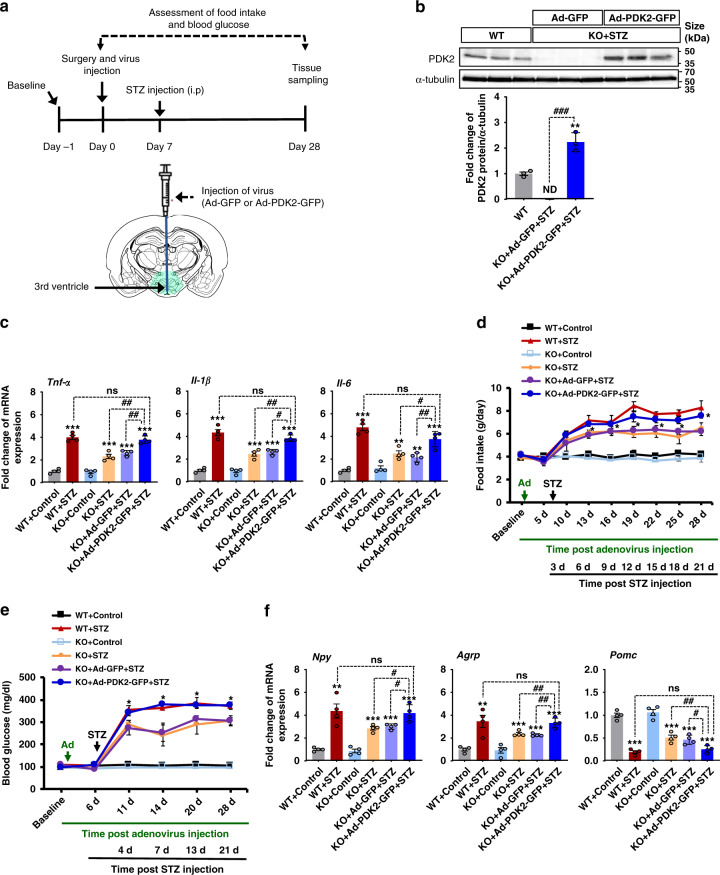
The effects of hypothalamic PDK2 overexpression on diabetes-induced inflammatory cytokines, food intake, blood glucose, and the expression of hypothalamic neuropeptides in *Pdk2* KO mice. To further determine the role of hypothalamic PDK2 in diabetes-induced neuroinflammation and dysregulation of feeding behavior, we administered Ad-PDK2-GFP or Ad-GFP (1.0 × 10^9^ pfu) via the icv route 1 w before STZ injection. The schematic diagram shows the experimentation timeline and the route of administration (**a**). PDK2 protein levels (###*p* = 4.5E-5, KO + Ad-PDK2-GFP + STZ) in the hypothalamus after 3 w of STZ and 4 w of adenovirus injection were assessed by Western blot analysis (**b**). Western blot band quantification for PDK2 protein levels normalized to α-tubulin. The relative expression of *Tnf-α* (##*p* = 0.0030, KO + Ad-PDK2-GFP + STZ), *Il-1β* (#*p* = 0.0380, KO + Ad-PDK2-GFP + STZ), and *Il-6* (##*p* = 0.0020, KO + Ad-PDK2-GFP + STZ) mRNA in the hypothalamic tissues isolated from Ad-PDK2-GFP or Ad-GFP and WT mice was evaluated by real-time RT-PCR (**c**). Food intake (*p = 0.0130, KO + Ad-PDK2-GFP + STZ at 28 d) (**d**) and fasting blood glucose levels (*p = 0.0127, KO + Ad-PDK2-GFP + STZ at 28 d) (**e**) were assessed following Ad-PDK2-GFP or Ad-GFP and STZ administration. Arrows indicate the time points of adenovirus (Ad) and STZ administration. The relative expression of *Npy* (#*p* = 0.0489, KO + Ad-PDK2-GFP + STZ), *Agrp* (##*p* = 0.0090, KO + Ad-PDK2-GFP + STZ), and *Pomc* (#p = 0.0270, KO + Ad-PDK2-GFP + STZ) mRNA in the hypothalamic tissues isolated from Ad-PDK2-GFP or Ad-GFP and STZ-injected mice just after completion of behavioral assessments was evaluated by real-time RT-PCR (**f**). Results for mRNA expression are displayed as the fold increase of mRNA expression normalized to *Gapdh*. *p < 0.05, **p < 0.01, or ***p < 0.001 versus the vehicle-treated control animals (**b**, **c**, **f**) or STZ/Ad-GFP + STZ-treated animals (**d**, **e**); #*p* < 0.05, ##*p* < 0.01 between the indicated groups. One-way ANOVA (**b**, **c**, **f**) and two-way ANOVA with Tukey’s post hoc test (**d**, **e**), *n* = 3 (b), *n* = 4 (**c**, **f**), and *n* = 6 (**d**, **e**); mean ± SEM. Value of “*n*” indicates the number of animals. Source data are provided as a Source data file. d day(s); STZ streptozotocin; pfu plaque-forming unit; ns not significant; ND not detected.

To further examine the role of hypothalamic PDK2 in diabetes-induced neuroinflammation and changes in feeding behavior, we assessed the effect of PDK2 overexpression in the *Pdk2* KO mice by injecting (icv) PDK2-overexpressing adenovirus (Ad-PDK2-GFP) into the third ventricle (Fig. 6a). Injection of Ad-PDK2-GFP into *Pdk2* KO mice enhanced PDK2 protein expression in the hypothalamus at 4 weeks post-Ad-PDK2-GFP administration (Fig. 6b). Remarkably, overexpression of hypothalamic PDK2 reversed the effects of *Pdk2* KO on diabetes-induced *Tnf-α, Il-1β*, and *Il-6* mRNA expression (Fig. 6c), food intake (Fig. 6d), blood glucose (Fig. 6e), and expression of *Npy, Agrp*, and *Pomc* in the diabetic hypothalamus at 3 weeks post-STZ injection (Fig. 6f). These findings further support that hypothalamic PDK2 plays a crucial role in diabetes-induced hypothalamic inflammation and subsequent dysregulation of feeding behavior.

### The PDK2-lactic acid axis modulates hypothalamic signaling involved in the orexigenic neuropeptide circuitry

**Fig. 7:**
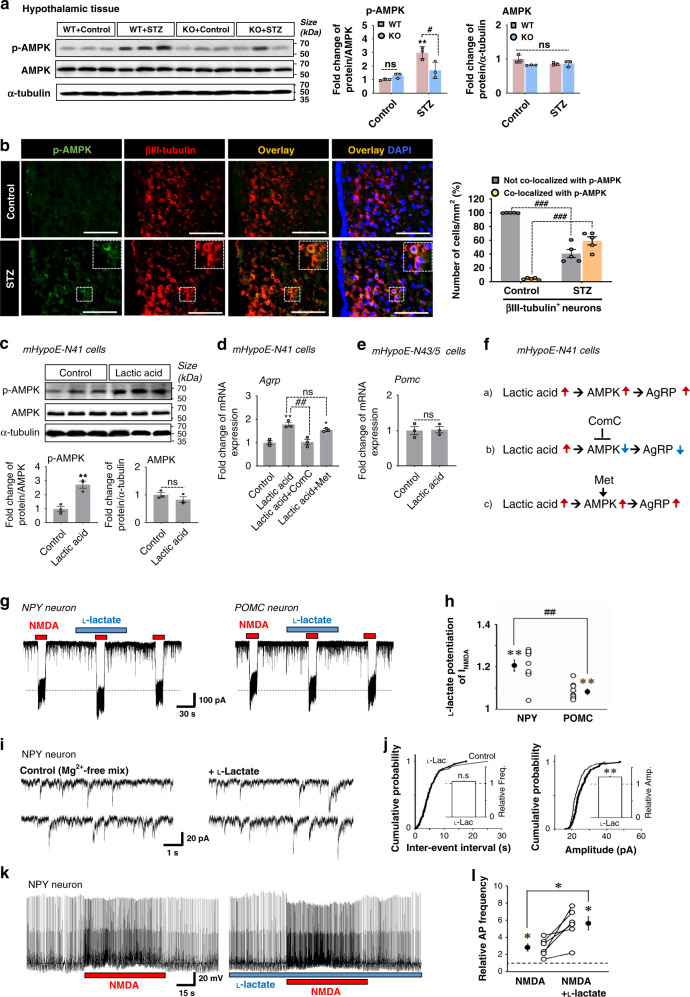
The role of PDK2 and lactic acid in regulating the AMPK signaling and food intake-related neuropeptide circuitry in diabetic mouse hypothalamus. p-AMPK (#*p* = 0.0157, KO + STZ) and AMPK protein levels in the hypothalamic tissues isolated from WT and *Pdk2* KO mice at 3 w post-STZ/vehicle injection were assessed by Western blot analysis (**a**). Co-immunofluorescence staining of mouse brain tissues showed p-AMPK and βIII-tubulin-positive neurons (###*p* = 1.4E-7, not co-localized with p-AMPK; and (###*p* = 3.7E-7, co-localized with p-AMPK) in the ARC at 3 w post-STZ/vehicle injection (**b**). The percentage of p-AMPK-positive neurons was calculated from the total number of cells present per mm^2^ using six randomly selected fields. Scale bar indicates 100 µm. The expression of p-AMPK (**p = 0.0051, lactic acid) and AMPK proteins (**c**), as well as *Agrp* (**d**) in mHypoE-N41 cells, and *Pomc* mRNA in mHypoE-N43/5 cells (**e**) after treating with lactic acid (7.1 mM) for 72 h was assessed by Western blot and real-time RT-PCR, respectively. The effect of Compound C (16 µM) and metformin (100 µM) on lactic acid-induced increased expression of *Agrp* mRNA (##*p* = 0.0011, lactic acid+ComC) in mHypoE-N41 cells was observed (**d**). Results for mRNA expression are displayed as the fold increase of gene expression normalized to *Gapdh*. The diagram shows the proposed lactic acid-AMPK axis in the regulation of the AgRP (**f**). Representative traces of *I*_NMDA_ induced by NMDA (100 μM) in the absence or presence of l-lactate (7.1 mM) in NPY/AgRP-expressing neurons (left) and POMC-expressing neurons (right) (**g**). l-lactate-induced changes of the NMDA-induced currents in NPY and POMC neurons (##*p* = 0.0006, POMC) (**h**). Representative traces of sEPSC_NMDA_ in the absence (left) or presence (right) of l-lactate in NPY neurons (**i**). Cumulative probability distribution for inter-event interval (left) and amplitude (right) of sEPSC_NMDA_ with or without l-lactate (**j**). The plot includes 195 events for control (thin lines) and 143 events for l-lactate (thick lines). Representative traces before, during, and after the application of NMDA in the absence (left) or presence (right) of l-lactate in NPY neurons in the current-clamp condition (**k**). NMDA-induced changes of action potential frequency in the absence or presence of l-lactate in NPY neurons (*p = 0.0220, NMDA + l-lactate) (**l**). Open and closed circles represent the value from individual neurons and their average respectively. Insets represent the l-lactate-induced changes in the frequency (left) and amplitude (right) of sEPSC_NMDA_. Dotted lines represent the relative control of basal frequency and amplitude of sEPSC_NMDA_. *p < 0.05, **p < 0.01, or ***p < 0.001 versus the vehicle-treated control animals/cells. Two-way ANOVA (**a**, **b**), one-way ANOVA with Tukey’s post hoc test (**d**, **h**), two-tailed Student’s *t*-test (**c**, **e**), two-tailed paired *t*-test (**h**, **j**, **l**), unpaired *t*-tes*t*, *n* = 9 individual neurons (**h**), *n* = 195 events for control and 143 for l-lactate (**j**), *n* = 6 individual neurons (**l**) *n* = 3 animals per group (**a**) or sister wells (**c**–**e**), and *n* = 5 animals per group (**b**); mean ± SEM. Source data are provided as a Source data file. ComC Compound C; Met metformin; L-lac l-lactate; Amp Amplitude; Freq Frequency; NC negative control; ns not significant.

AMPK acts as a cellular energy sensor, and it is expressed in the hypothalamic neurons involved in regulating feeding behavior^40^. Uncontrolled diabetes in rodents increases hypothalamic AMPK activity as indicated by higher levels of phosphorylated-AMPK (p-AMPK), which leads to increased food intake^41^. Consistent with this finding, increased levels of the p-AMPK protein were detected in the hypothalamus of diabetic mice at 3 weeks post-STZ treatment. Interestingly, *Pdk2*-deficient mice exhibited lower levels of the p-AMPK protein in diabetic hypothalamus when compared to WT-diabetic animals (Fig. 7a). Immunofluorescence staining and image analysis of brain tissue sections isolated from diabetic mice revealed that p-AMPK is predominantly increased in hypothalamic neurons. This was confirmed by double labeling with βIII-tubulin (Fig. 7b).

Enhanced expression of PDK2 and augmented lactic acid production in diabetic mouse hypothalamus led us to investigate the mechanistic relationship between the PDK2-lactic acid axis and AMPK activity in orexigenic neurons, which might be involved in the diabetes-induced alterations in feeding behavior. We compared lactic acid responses in cultured hypothalamic cells expressing NPY, AgRP, or POMC neuropeptides. Exposure of NPY/AgRP-expressing mHypoE-N41 cells to lactic acid (7.1 mM) for 72 h significantly increased the levels of p-AMPK protein (Fig. 7c) and *Agrp* mRNA (Fig. 7d). However, lactic acid did not alter the expression level of *Pomc* mRNA in mHypoE-N43/5 cells, embryonic mouse hypothalamic cells expressing POMC (Fig. 7e). We co-treated mHypoE-N41 cells with lactic acid and a small-molecule inhibitor (Compound C, 16 µM) or an activator (metformin, 100 mM) of AMPK. The resulting data revealed that Compound C, but not metformin exposure, inhibits lactic acid-induced expression of *Agrp* mRNA in mHypoE-N41 cells (Fig. 7d). This suggests that AMPK plays a crucial role in modulating lactic acid-induced expression of AgRP neuropeptide in diabetic hypothalamus (Fig. 7f). Moreover, the hypothalamic AKT pathway is also known to regulate appetite. Activation of this pathway in NPY/AgRP neurons also suppresses appetite by reducing the synthesis of NPY/AgRP peptides^42^. In the present study, substantially lower levels of phosphorylated-AKT (p-AKT) protein were detected in the hypothalamus of diabetic mice (Supplementary Fig. 3a). However, *Pdk2*-deficiency reversed the reduction in p-AKT protein levels in diabetic hypothalamus (Supplementary Fig. 3a). Next, we determined whether lactic acid influences the AKT signaling pathway. Exposing mHypoE-N41 cells to lactic acid for 30 min decreased the levels of p-AKT (Supplementary Fig. 3b). Consistently, a negative correlation between AMPK and AKT activation in the hypothalamus has been reported with respect to the regulation of feeding behavior of rats through modulation of NPY expression^43^. These findings suggest that the PDK2-lactic acid axis plays a pivotal role in the dysregulation of AMPK and AKT signaling and the associated orexigenic neuropeptidergic circuit in the hypothalamus. This likely accounts for the increased food intake seen in diabetic mice.

Additionally, we investigated the effect of lactate on NPY/AgRP and POMC neuronal activity using whole-cell patch-clamp and single-cell RT-PCR techniques. Single ARC neurons were isolated from the hypothalamic region of mouse brain tissue slices, and the effects of lactate on NMDA receptors were examined using a whole-cell patch-clamp technique. Identity of ARC neurons was confirmed after electrophysiological recordings by using single-cell RT-PCR analysis to detect transcripts for NPY and POMC (Supplementary Fig. 3e). Exposure of NPY-expressing neurons to l-lactate (7.1 mM) significantly increased NMDA (100 μM)-induced membrane currents (120.6 ± 2.6% of the control, *n* = 9, *p* < 0.01, Fig. 7g, h). l-lactate (7.1 mM) also potentiated the NMDA-induced currents in POMC-expressing neurons (108.2 ± 1.3% of the control, *n* = 9, *p* < 0.01), but the extent of potentiation was significantly lower than in NPY-expressing neuron (*p* < 0.01, Fig. 7g, h). Although the reason for this discrepancy remains to be elucidated, the present results suggest that l-lactate may selectively alter the orexigenic circuit. In contrast to l-lactate, d-lactate (7.1 mM), which was used as a control^44,45^, had no effect on NMDA-induced (100 μM)-membrane currents in both NPY-expressing and POMC-expressing neurons (Supplementary Fig. 3c, d). In order to examine whether l-lactate affects synaptic NMDA receptors, spontaneous excitatory postsynaptic currents mediated by NMDA receptors (sEPSC_NMDA_) were recorded at a holding potential of −60 mV in the Mg^2+^-free external solution containing 20 μM CNQX, 1 μM strychnine, and 10 μM glycine (Mg^2+^-free mix; Supplementary Fig. 3f, g). l-lactate (7.1 mM) increased the amplitude of spontaneous excitatory postsynaptic currents mediated by NMDA receptors to 121.2 ± 3.2% of the control (*n* = 7, *p* < 0.01) without affecting their frequency (105.9 ± 2.2% of the control, *n* = 7, *p* = 0.12, Fig. 7i, j). In addition, l-lactate shifted the cumulative distribution of the amplitude of sEPSC_NMDA_ (p < 0.01, K–S test) without affecting the cumulative distribution of frequency (*p* = 0.49, K–S test), consistent with an increase in the amplitude of synaptic NMDA receptors. Next, we directly examined whether l-lactate changes the excitability of NPY/AgRP-expressing neurons in the current-clamp recording. We found that NMDA exerted greater effects on the frequency of action potentials in the presence of 7.1 mM l-lactate (550.3 ± 77.3% of the control, *n* = 6, *p* < 0.05) than in the absence of l-lactate (282.6 ± 2.6% of the control, *n* = 6) (Fig. 7k, l). Collectively, it is suggested that NPY/AgRP neurons are more susceptible to l-lactate than the POMC neurons in the hypothalamic ARC, conditions that can be associated with diabetes-induced alterations in feeding behavior.

### Selective deletion of *Pdk2* from hypothalamic astrocytes attenuates diabetes-induced local neuroinflammation and increased food intake

**Fig. 8:**
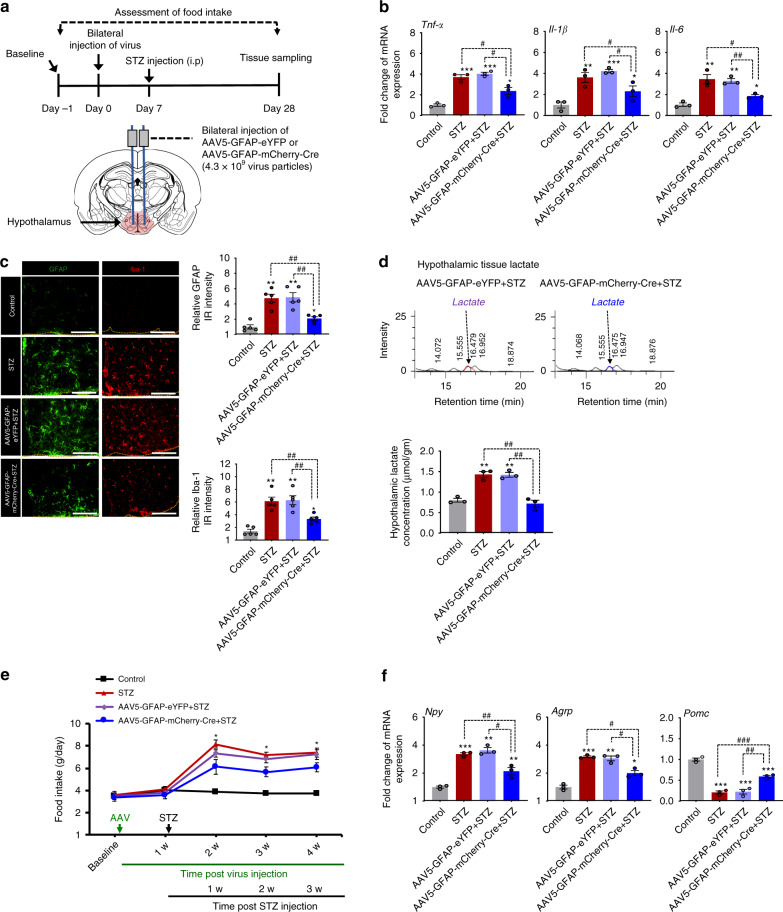
Effects of astrocyte-specific Cre-mediated *Pdk2* gene ablation on diabetes-induced hypothalamic inflammation and changes in feeding behavior. To determine the role of hypothalamic PDK2 in the regulation of neuroinflammation and feeding behavior, we administered AAV5-GFAP-mCherry-Cre or AAV5-GFAP-eYFP (4.3 × 10^9^ virus particles) into the mediobasal hypothalamus of *Pdk2* floxed mice, bilaterally, using a stereotaxic device 1 w before STZ injection. The schematic diagram presents the experiment timeline and route of administration (**a**). The relative expression of *Tnf-α* (#*p* = 0.0141, AAV5-GFAP-mCherry-Cre+STZ), *IL-1β* (#*p* = 0.0316, AAV5-GFAP-mCherry-Cre+STZ), and *IL-6* (##*p* = 0.0016, AAV5-GFAP-mCherry-Cre+STZ) mRNA in the hypothalamic tissues isolated from AAV5-GFAP-mCherry-Cre or AAV5-GFAP-eYFP and STZ-injected mice was evaluated by real-time RT-PCR (**b**). GFAP (##*p* = 0.0020, AAV5-GFAP-mCherry-Cre + STZ) and Iba-1 (##*p* = 0.0053, AAV5-GFAP-mCherry-Cre+STZ) immunofluorescence staining revealed their increased immunoreactivity in the hypothalamus of diabetes *Pdk2* floxed mice, but *Pdk2*-deficiency in hypothalamic astrocytes attenuated such an increase in the immunoreactivities at 3 w post-STZ injection (**c**). Microscope data were gathered using five randomly selected fields captured at the same magnification. Scale bar indicates 200 µm. Lactate concentration (##*p* = 0.0030, AAV5-GFAP-mCherry-Cre+STZ) in hypothalamic tissues collected from AAV5-GFAP-mCherry-Cre- or AAV5-GFAP-eYFP- and STZ-injected mice was measured with an HPLC analyzer (**d**). Quantification of the lactate concentration from HPLC graphs is shown. Food intake (*p = 0.0393, AAV5-GFAP-mCherry-Cre+STZ at 4 w) was assessed following AAV5-GFAP-mCherry-Cre or AAV5-GFAP-eYFP and STZ administration (**e**). Arrows indicate the time points of adeno-associated virus (AAV), and STZ administration. The relative expression of *Npy* (#*p* = 0.0140, AAV5-GFAP-mCherry-Cre+STZ), *Agrp* (#*p* = 0.0380, AAV5-GFAP-mCherry-Cre+STZ) and *Pomc* (##*p* = 0.0090, AAV5-GFAP-mCherry-Cre + STZ) mRNA in hypothalamic tissues isolated from AAV5-GFAP-mCherry-Cre- or AAV5-GFAP-eYFP- and STZ-injected mice just after completion of behavioral assessments was evaluated by real-time RT-PCR (**f**). mRNA expression results are displayed as the fold increase of mRNA expression normalized to *Gapdh*. *p < 0.05, **p < 0.01, or ***p < 0.001 versus the vehicle-treated control animals (**b**–**d**, **f**) or STZ/AAV5-GFAP-eYFP+STZ-treated animals (**e**); #*p* < 0.05, ##*p* < 0.01, or ###*p* < 0.001 versus indicated groups. One-way ANOVA (**b**–**d**, **f**) and two-way ANOVA (**e**) with Tukey’s post hoc test, *n* = 3 (**b**, **d**, **f**), *n* = 5 (**c**), and *n* = 6 (**e**); mean ± SEM. Value of “*n*” indicates the number of animals. Source data are provided as a Source data file. w week(s); IR immunoreactivity.

PDK2 is expressed at high levels in both hypothalamic astrocytes and neurons following diabetes. To determine the role of PDK2 in hypothalamic astrocytes and neurons in diabetes-induced inflammation and altered feeding behavior, we first generated mice in which the *Pdk2* gene was selectively inactivated in hypothalamic astrocytes by bilateral injection of AAV5-GFAP-mCherry-Cre into the hypothalamus of homozygous *Pdk2* floxed mice. Immunofluorescence staining of brain tissues from virus-infected animals showed a majority of co-localized mCherry- and GFAP-positive cells but not NeuN-positive neurons or Iba-1-positive microglia in the mice hypothalamus at 4 weeks after AAV5-GFAP-mCherry-Cre or AAV5-GFAP-eYFP injection (Supplementary Fig. 4a). The efficiency and specificity of GFAP-Cre-mediated recombination were confirmed by analyses of mCherry- or eYFP-positive cells isolated from hypothalamic tissues by digestion and FACS sorting (Supplementary Fig. 4b). RT-PCR analysis of FACS-sorted cells confirmed a successful deletion of the *Pdk2* gene from hypothalamic astrocytes (Supplementary Fig. 4c). Remarkably, ablation of astrocytic *Pdk2* in the hypothalamus attenuated diabetes-induced local inflammation, characterized by increased levels of the inflammatory cytokines *Tnf-α, Il-1β*, and *Il-6* mRNA (Fig. 8b), and proliferation and activation of GFAP-positive astrocytes and Iba-1-positive microglia (Fig. 8c). In addition, mice with selective deletion of *Pdk2* in hypothalamic astrocytes showed lower levels of diabetes-induced food intake (Fig. 8e), lactate surge (Fig. 8d), and *Npy* and *Agrp* mRNAs but higher levels of *Pomc* mRNA (Fig. 8f) in the hypothalamus when compared with the AAV5-GFAP-eYFP-injected diabetic animals. This indicates that astrocyte-specific PDK2 plays a critical role in hypothalamic inflammation and subsequent dysregulation of neuropeptidergic circuitry associated with altered feeding behavior in diabetes.

Next, we used mice in which the *Pdk2* gene was selectively ablated in hypothalamic neurons, generated by the AAV2-hSyn-mCherry-Cre-mediated *Pdk2* recombination approach (Supplementary Fig. 5a). Immunostaining and RT-PCR data confirmed a successful deletion of the *Pdk2* gene from hypothalamic neurons (Supplementary Fig. 5b–d). Ablation of neuronal *Pdk2* in the hypothalamus did not alter diabetes-induced expression levels of *Tnf-α, Il-1β*, and *Il-6* mRNA (Supplementary Fig. 5e), or gliosis (Supplementary Fig. 5f). However, mice with selective deletion of *Pdk2* in hypothalamic neurons showed a remarkable reduction in diabetes-induced food intake (Supplementary Fig. 5g). These findings suggest that although neuronal PDK2 potentially contributes to diabetes-induced altered feeding behavior, this molecule seems to be more involved in astrocytic than neuronal inflammatory pathways underlying these disorders. Future studies are required to better understand the role of hypothalamic neuronal PDK2 in the regulation of feeding behavior associated with diabetes, but these findings further support our claim that PDK2 in hypothalamic astrocytes is crucial for hypothalamic inflammation and subsequent dysregulation of neuropeptidergic circuitry associated with altered feeding behavior in diabetes.

## Discussion

Our findings demonstrate a critical role that astrocyte-specific PDK2 plays in the hypothalamus in regulating metabolic and inflammatory pathways that contribute to the hypothalamic pathology of altered feeding behavior associated with diabetes. Our data implicate lactic acid as an inflammatory mediator and a signaling molecule in diabetes. In addition, the PDK2-lactic acid axis modulates hypothalamic signaling involved in the regulation of the neuropeptide circuitry associated with feeding behavior in diabetic mice (Supplementary Fig. 8f).

Following the onset of diabetes, the expression of PDK2 and p-PDH was significantly up-regulated in mouse hypothalamus and limited to the ARC region. The unique PDK2 expression pattern in the hypothalamus might correlate with hypothalamic pathology observed in the early phase of metabolic diseases, including obesity and diabetes^5^. Increasing amounts of evidence suggest that hypothalamic inflammation, particularly in the ARC, is a common pathological feature related to obesity and type-2 diabetes^46^. In addition, microglia and astrocytes with inflammatory activation play crucial roles in the hypothalamic pathophysiology of these diseases^47^. However, very little is known about the function of glial cells in type 1 diabetes. Higher levels of hypothalamic microglia and astrocyte activation and proliferation have been observed in STZ-induced type 1 diabetic rodents, as has augmented expression of IFN-γ and IL-1β^23,24^. Our mouse model studies reveal that inhibition of hypothalamic inflammation significantly reduces diabetes-induced hypothalamic pathology involved in the alteration of feeding behavior. This finding suggests that hypothalamic inflammation is crucial in type 1 diabetes. We also observed that genetic deletion or pharmacological inhibition of hypothalamic PDK2 significantly reduced diabetes-induced inflammatory markers and food intake associated with an increase in orexigenic neuropeptide and decreased anorexigenic neuropeptide expression. This suggests that PDK2 plays a pivotal role in hypothalamic inflammation and subsequent pathology of altered feeding behavior in diabetes.

PDK2 is predominantly expressed in astrocytes and neurons but not microglia, indicating that astrocytes are one of the major cell types that express PDK2 in the ARC of diabetic mice. Astrocytes are primarily glycolytic, demonstrating a lower oxidative metabolic rate when compared to neurons^48^. The higher expression of PDK2 in astrocytes, when compared to neurons^49^, is consistent with the higher levels of p-PDH and lactate production as an end product of glycolysis displayed by astrocytes^33,50^. Most of the glucose entering the glycolytic pathway in astrocytes is released as lactate in the extracellular space^51^. PDK is a crucial regulator of glycolytic reprogramming in macrophages and causes them to shift to an inflammatory M1 phenotype^35^. However, the role of PDK2 in glycolytic metabolism and inflammatory activation of astrocytes has not been revealed. Our in vitro and in situ studies using primary astrocytes and hypothalamic tissues isolated from *Pdk2* KO mice showed that PDK2 plays a critical role in the high glucose-induced alteration of glycolytic metabolism and the inflammatory activation of astrocytes that is accompanied by increased levels of inflammatory cytokines and lactate surge. Moreover, pharmacological inhibition of astrocytic lactic acid production suppresses high-glucose-induced inflammatory cytokine gene expression, suggesting a vital role of the PDK2-lactic acid axis in the inflammatory activation of astrocytes in the diabetic hypothalamus.

PDK2 modulates astrocyte metabolism and leads to their inflammatory activation. This drives neuronal dysfunction in the hypothalamus, which is involved in altered feeding behavior following the onset of diabetes. This was discovered using hypothalamic astrocyte-specific *Pdk2* KO mice. These mice had reduced inflammatory activation of astrocytes, expression levels of orexigenic neuropeptides in the hypothalamus, and food intake after acquiring diabetes. Congruent with our results, a recent study demonstrated that chemogenetic activation of astrocytes in the ARC via the application of designer drugs enhances the activity of NPY/AgRP-expressing neurons without affecting POMC-expressing neurons, which increases feeding in mice^22^.

PDK2 is also expressed in hypothalamic neurons following diabetes induction. Neurons also produce lactate upon stimulation/excitation^48,52^. A study by Walz et al. revealed that primary cerebral neurons isolated from mice stimulated by dinitrophenol, adenosine, and insulin show increased release of lactate in culture^48^. In the present study, the onset of diabetes increased the level of lactate in the hypothalamus, but lactate was significantly decreased in *Pdk2*-deficient animals, suggesting that increased expression of neuronal PDK2 might also contribute to accumulated lactate in the diabetic hypothalamus. However, our study using hypothalamic astrocyte and neuron-specific *Pdk2* KO mice indicated that astrocytic PDK2 and ensuing lactic acid production play a neuroinflammatory role in the diabetic hypothalamus. In addition, a substantial increase in lactate has also been observed in the hypothalamus of obese mice fed with HFD^53^. Patients with diabetes frequently exhibit increased blood lactate levels with decreased pH^54^. Increased lactate with decreased pH might serve as a potential inducer of proinflammatory cytokine production in the hypothalamus. A lactic acid-induced acidic microenvironment has been reported to provoke the activation of microglia and astrocytes in culture, which allows them to release diverse proinflammatory mediators, suggesting that lactic acid is a critical inflammatory mediator^55^. These findings led us to speculate that the lactic acid produced from astrocytes may further amplify the activation of microglia and contribute to hypothalamic inflammation. The interaction between these two glial cell types strongly influences hypothalamic inflammation and the progression of neurometabolic diseases^56^. Interglial communication occurs through different cell surface molecules or through the release of signaling molecules such as cytokines, chemokines, and adenosine triphosphate^57^. In addition, these glia-derived molecules also have a neuromodulatory role, and a few of them are termed gliotransmitters^58^. Similarly, lactate has been considered a signaling molecule or gliotransmitter in several studies^44,50^. Accordingly, lactate has been recently implicated in neuronal plasticity^45^, neuron-glia interactions^44^, neuroimmune communication, and nociception^35^. Our findings imply that lactic acid derived from astrocytes in the hypothalamus may function as both an inflammatory mediator and a signaling molecule that modulates the neuronal circuitry associated with altered feeding behavior in diabetes.

STZ-induced diabetic mice showed higher levels of p-AMPK in the hypothalamus, indicating increased AMPK activation, particularly in hypothalamic neurons, which was attenuated by *Pdk2* deficiency. AMPK is a serine/threonine protein kinase that is activated when cellular energy is depleted; thus, it acts as the cellular energy sensor of the hypothalamus^59^. AMPK expressed in hypothalamic neurons is involved in the regulation of food intake by controlling the level of appetite-related neuropeptides in the melanocortin system^40^. Greater APMK activity has been observed in the hypothalamus of diabetic rats, and inhibition of this activity prevents diabetes-induced increased food intake in rats^41^. In addition, central lactate metabolism has been reported to regulate food intake and plasma glucose levels in rodents; exogenous lactate administration suppressed food intake acutely through modulation of hypothalamic AMPK/malonyl-CoA signaling in rats under physiological conditions^60^. However, chronic effects of higher levels of endogenous lactate or lactic acidosis and underlying molecular and cellular mechanisms under pathological conditions like diabetes have not been investigated. Interestingly, our in vitro study revealed that treating hypothalamic AgRP-expressing cells with lactic acid increased the expression of p-AMPK protein and *Agrp* mRNA. This finding is also supported by a previous study in which the chronic exposure of mouse primary neurons and neuroblastoma cells to lactic acid increased the expression of p-AMPK protein^61^. Taken together, these results suggest that the PDK2-lactic acid axis may play a crucial role in the dysregulation of the appetite-regulating hypothalamic signaling and neuropeptidergic circuits involved in diabetic feeding behavior.

Electrophysiology studies of mouse brain slice cultures reveal that lactate can increase the excitability of ARC neurons by potentiating excitatory NMDA receptors. The effect of lactate on neuronal excitability was reported previously^62^. That study demonstrated that lactate is necessary for maintaining the spontaneous activity of orexin neurons in hypothalamic slices through a mechanism involving ATP-dependent potassium channels. Similarly, another study found that locus coeruleus noradrenergic neurons are hyperexcited by increased lactate released from activated astrocytes^44^. This effect seems to depend on an as yet unidentified “lactate receptor” that is positively coupled to cAMP formation. These findings suggest that lactate is released primarily by astrocytes, which then modulates neuronal activity. Furthermore, an optogenetic study revealed that stimulation of AgRP neurons produces inhibitory postsynaptic currents in POMC neurons, leading to the inhibition of POMC neuron firing^63^. A recent study found that optogenetic activation of AgRP neurons in the mouse hypothalamus increased food intake in an appetite-suppressing condition induced by the administration of amylin, cholecystokinin, and lithium chloride^64^. In our study, NPY/AgRP-expressing neurons showed a higher excitability rate than the POMC-expressing neurons in the presence of lactate, suggesting that NPY/AgRP-expressing neurons are more susceptible to lactate and that lactate-induced excitation of POMC neurons may not be sufficient to suppress NPY/AgRP neuronal activity. Taken together, these findings led us to speculate that enhanced expression of PDK2 in astrocytes and a subsequent release of lactic acid in the hypothalamus profoundly up-regulate orexigenic neuronal activity in the ARC, thereby causing a substantial increase in food intake in diabetic mice.

In conclusion, our findings suggest that astrocytic PDK2 plays an important role in hypothalamic inflammation and consequent impairment of feeding behavior in diabetes. A hyperglycemia-induced increase in the expression of PDK2 and phosphorylation of PDH leads to a glycolytic shift in the diabetic hypothalamus. The PDK2-mediated metabolic shift from oxidative phosphorylation to glycolysis in astrocytes contributes to neuroinflammatory responses in the diabetic hypothalamus. Moreover, the augmented lactic acid production adversely affects appetite-regulating neuronal function. Our findings identify the PDK2-lactic acid axis in hypothalamic astrocytes as a promising therapeutic target for pathological hypothalamic inflammation and subsequent dysregulation of the neuropeptidergic circuit involved in altered feeding behavior in diabetes.

## Methods

### Mouse breeding and maintenance

All experiments were conducted in accordance with approved animal protocols and guidelines established by the Animal Care Committee of Kyungpook National University (Approval No., KNU-2012-73/66). Male wild-type (WT) and *Pdk2* knockout (KO) mice^65^ aged 8–10 weeks were used. Age-matched WT mice were produced from the C57BL/6J mice (The Jackson Laboratory, Bar Harbor, ME), which were used to stabilize the genetic backgrounds of the *Pdk2* KO. Animals were housed under a 12-hr light/dark cycle (lights on 07:00–19:00) at a constant ambient temperature of 23 ± 2 °C with food and water provided ad libitum. Each individual animal was used for a single experimental purpose.

### Streptozotocin-induced diabetes models

The mouse model of uncontrolled diabetes was generated using two experimental protocols. First, typical type 1 diabetes was induced by a single intraperitoneal injection of high-dose streptozotocin (STZ, Sigma-Aldrich, St. Louis, MO; 150 mg/kg body weight) in 0.1 M citrate buffer (pH 4.5). Second, mild type 1 diabetes was induced by intraperitoneal injection of multiple low-doses of STZ (MLDS, 40 mg/kg body weight) in 0.1 M citrate buffer (pH 4.5) for 5 consecutive days. Blood samples were collected from the tail vein after 3 days of single STZ injection and 7 days of MLDS injections, and glycemia was determined by using SD CodeFree^TM^ glucometer (SD Biosensor Inc., Korea). Animals with fasting blood glucose values higher than 260 mg/dl were considered diabetic.

### High-fat diet model

Six-week-old male WT (C57BL/6J) and *Pdk2* KO mice were fed an HFD in which 20% of the calories were derived from carbohydrates and 60% from fat (D12492 pellets; Research Diets). Control WT and *Pdk2* KO mice were fed an isocaloric low-fat diet/control diet (CD) in which 70% of the calories were derived from carbohydrates and 10% from fat (D12450B pellets; Research Diet). The mice were housed and maintained on a 12-h light/dark cycle at 22 ± 2 °C. After 16 weeks, all HFD-fed WT mice showed significant weight gain and elevated fasting blood glucose levels when compared with CD-fed mice.

### Primary astrocyte cultures

For primary astrocyte cultures, the brains of 3-day-old mice were homogenized and mechanically disrupted with a nylon mesh. The obtained mixed glial cells were seeded in culture flasks and grown at 37^o^C in a 5% CO_2_ incubator in Dulbecco’s Modified Eagle’s medium (DMEM) supplemented with 5.5 mM glucose (low glucose DMEM, Hyclone Laboratories Inc.), 10% FBS, 100 U/ml of penicillin, and 100 μg/ml of streptomycin (Gibco, Grand Island, NY). Culture media were initially changed after 5 days and then changed every 3 days. After 14 days, the mixed glial culture was mechanically agitated at 200 cycles per minute overnight. The culture media containing cells that were detached from the substratum was discarded; astrocytes were dissociated using trypsin–EDTA (Invitrogen) and then collected by centrifugation at 282 × *g* for 10 min. The collected primary astrocytes were cultured in DMEM supplemented with 5.5 mM glucose, 10% FBS, and penicillin-streptomycin.

### Organotypic hypothalamus slice cultures

Brain tissues were isolated from *Pdk2* floxed or AAV5-GFAP-eYFP or AAV5-GFAP-mCherry-Cre-injected 7-8-week-old mice and sectioned transversely at 300 μm with a microslicer (VT1000S; Leica, Nussloch, Germany) under sterile conditions. The tissues from hypothalamic area were dissected manually and plated onto Millipore-Millicell-CM mesh inserts (Fisher Scientific) in six-well culture plates at four explants per insert. The culture was maintained with DMEM supplemented with 5.5 mM glucose, 1% Glutamax (Gibco), 10% FBS, 100 U/ml of penicillin, and 100 μg/ml of streptomycin in an incubator at 5% CO_2_ and 37 °C. The culture medium (1 ml) was replaced at 2-day-intervals up to 10 days. Then, the tissues were treated with high glucose concentration (25 mM) to determine the role of astrocytic PDK2 on high glucose-induced changes in the expression levels of inflammatory cytokines.

### Assessment of food intake

Mice were caged individually and allowed at least 3 days for adaptation. A known (i.e., pre-weighed) amount of food was placed in their home cage hopper and the remaining food was weighed after 24 h following the light-dark cycles. Similarly, 12-h food intake was assessed following dark cycles. Extra precautions were taken to minimize the usual errors due to spillage or hoarding behavior. Specifically, broken and very small visible food particles were collected from the bedding and the weights were corrected to minimize errors. The bedding was changed every test day just before the test. For the HFD food intake assessment, food pellets were compacted with manual finger pressure to minimize their breakability on movement. Calorie intake was calculated based on Kcal per gram of food (collected information from company data sheets, HFD, D12492 pellets; Research Diets; CD, D12450B pellets; Research Diet).

### Thermogenesis assay

After 16 weeks’ post-HFD/CD feeding, mice were individually housed at 4 °C, and their rectal temperature was measured every 2 h over the course of 6 h using a digital thermometer (Temperature Control Unit HB 101/2; Panlab, Harvard Apparatus).

### Quantitative real-time and traditional reverse transcription-PCR

Real-time RT-PCR was performed using the one-step SYBR^®^ PrimeScript^TM^ RT-PCR kit (Perfect Real-Time; Takara Bio Inc., Tokyo) and the ABI Prism^®^ 7000 sequence detection system (Applied Biosystems, Foster City, CA), according to the manufacturer’s instructions. The 2^−ΔΔCt^ method was used to calculate the relative changes in gene expression, and *Gapdh* was used as an internal control. The nucleotide sequences of the primers used in the real-time RT-PCR were as follows: *Pdk1*, forward, 5′-CAC CAC GCG GAC AAA GG-3′, reverse, 5′-GCC CAG CGT GAC GTG AA-3′; *Pdk2*, forward, 5′-CCC CGT CCC CGT TGT C-3′, reverse, 5′-TCG CAG GCA TTG CTG GAT-3′; *Pdk3*, forward, 5′-GGA GCA ATC CCA GCA GTG AA-3′, reverse, 5′-TGA TCT TGT CCT GTT TAG CCT TGT-3′; *Pdk4*, forward, 5′-CCA TGA GAA GAG CCC AGA AGA-3′, reverse, 5′-GAA CTT TGA CCA GCG TGT CTA CAA-3′; *Tnf-α*, forward, 5′-ATG GCC TCC TCA TCA GTT C-3′, reverse, 5′-TTG GTT TGC TAC GAC GTG-3′; *Il-1β*, forward, 5′-AAG TTG ACG GAC CCC AAA AGA T-3′, reverse, 5′-TGT TGA TGT GCT GCT GCG A-3′; *Il-6*, forward, 5′-AGT TGC CTT CTT GGG ACT GA-3′, reverse, 5′-TCC ACG ATT TCC CAG AGA AC-3′; *Npy*, forward, 5′-CTA CTC CGC TCT GCG ACA CT-3′, reverse, 5′-AGT GTC TCA GGG CTG GAT CTC-3′; *Agrp*, forward, 5′-CGG CCA CGA ACC TCT GTA G-3′, reverse, 5′-CTC ATC CCC TGC CTT TGC-3′; *Pomc*, forward, 5′-GAG GCC ACT GAA CAT CTT TGT C-3′, reverse, 5′-GCA GAG GCA AAC AAG ATT GG-3′; and *Gapdh*, forward, 5′-TGG GCT ACA CTG AGC ACC AG-3′, reverse, 5′-GGG TGT CGC TGT TGA AGT CA-3′. The nucleotide sequences of the primers used in the traditional-RT-PCR were as follows: *Pdk2*, forward, 5′- GTC TGC TGG ACA TCA TGG AAT-3′, reverse, 5′-CAT AGG CGT CTT TCA CCA CAT-3′; *Gfap*, forward, 5′-AAG CAG AAG CTC CAA GAT GA-3′, reverse, 5′-GAG GTC TGC AAA CTT GG-3′; *Iba-1*, forward, 5′-GAA GCG AAT GCT GGA GAA-3′, reverse, 5′-GAC CAG TTG GCC TCT TGT-3′; *Tubb3*, forward, 5′-CAA TGC CGA CCT CCG CAA GC-3′, reverse, 5′-ATG AAG CAC CTC ACC TAG GG-3′; *Pparβ/δ*, forward, 5′-GGC CAT GGG TGA CGG AGC-3′, reverse, 5′-GAT CTT GCA GAT CCG ATC GC-3′; and *Gapdh*, forward, 5′-ACC ACA GTC CAT GCC ATC AC-3′, reverse, 5′-TCC ACC ACC CTG TTG CTG TA-3′.

### Western blot analysis

Protein was prepared from mouse hypothalamic tissues or cells and the concentration was determined with a Pierce^TM^ BCA Protein Assay Kit using bovine serum albumin as the standard. Proteins (20–30 μg) from each sample were separated on 12% or 15% SDS-PAGE gels and transferred to PVDF membranes (Bio-Rad, Hercules, CA) by the semidry electroblotting method. The membranes were blocked with 5% skim milk and sequentially incubated with primary antibodies against PDK1 (rabbit, 1:1000; Enzo Life Sciences), PDK2 (rabbit, 1:1000; Abgent, San Diego, CA), PDK3 (rabbit, 1:1000; Abnova), PDK4 (rabbit, 1:1000; Atlas Antibodies AB), phospho-Ser293-PDH-E1α (pyruvate dehydrogenase E1α) (rabbit, 1:1000; Calbiochem), phospho-Ser300-PDH-E1α (pyruvate dehydrogenase E1α) (rabbit, 1:1000; Calbiochem), PDH-E1 (rabbit, 1:1000; Cell Signaling), phosphorylated (Thr-172) and total forms of AMPKα (rabbit, 1:1000; Cell Signaling Technology), phosphorylated (Ser473) and total forms of AKT (rabbit, 1:1000; Cell Signaling Technology) or α-tubulin (mouse, 1:2000; Sigma-Aldrich), and horseradish peroxidase-conjugated secondary antibodies (anti-rabbit or mouse IgG antibody; Cell Signaling Technology), followed by enhanced chemiluminescence detection (Thermo Fisher Scientific).

### Immunohistochemistry

Mice were deeply anesthetized and then perfused through the aorta with 0.1 M phosphate-buffered saline (PBS) followed by 4% paraformaldehyde (PFA) fixative. Whole brain tissue was collected, post-fixed in the same PFA fixative overnight, and cryoprotected with 30% sucrose in 0.1 M PBS overnight at 4°C. A cryostat was used to prepare 20-µm-thick coronal tissue sections. The sectioned brain tissues were mounted on gelatin-coated slides and tissue sections were placed in 0.1 M PBS. Sections were then blocked with 4% normal donkey serum in 0.3% Triton X-100 for 60 min at room temperature. For immunofluorescence or 3, 3′-diaminobenzidine (DAB) staining, sections were incubated with primary antibodies against PDK2 (rabbit, 1:100; Abgent, San Diego, CA), Iba-1 (goat, 1:200; Novus Biologicals, Littleton, CO) or Iba-1 (rabbit, 1:1000; Wako Pure Chemical Corporation), GFAP (mouse, 1:200; Novus Biologicals, Littleton, CO) or GFAP (rabbit, 1.500; DAKO), βIII-tubulin (mouse, 1:200; Santa Cruz Biotechnology Inc.), NeuN (rabbit, 1:500, Merck Millipore), AGRP (rabbit, 1:200; Phoenix Pharmaceuticals Inc.), POMC (rabbit, 1:200, phoenix Pharmaceutical Inc.), NF-κB p65 (mouse, 1:200, Santa Cruz Biotechnology Inc), phosphorylated (Thr-172) and total forms of AMPKα (rabbit, 1:100; Cell Signaling Technology) overnight at 4°C. For immunofluorescence staining, sections were incubated with FITC- or Cy3 or Cy5-conjugated secondary antibodies (1:200; Jackson ImmunoResearch, West Grove, PA). Slides were washed, coverslipped with Vectashield mounting medium (Vector Laboratories, Burlingame, CA), and visualized under a fluorescence (Leica Microsystems, DM2500, Wetzlar, Germany). For DAB staining, the sections were incubated with biotin-conjugated secondary antibodies for 1 hr at room temperature, and the signal was detected by using an avidin-biotin complex kit (Vector Laboratories) and DAB kit (Vector Laboratories).

### Enzyme-linked immunosorbent assay

A mouse insulin Enzyme-linked immunosorbent assay (ELISA) kit (ALPCO), mouse/rat leptin ELISA kit (R&D Systems), and mouse TNF-α kit (R&D Systems) were used to assess the concentration of insulin, leptin, and TNF-α respectively by following the protocol as described in the provided product manuals.

### Measurement of ketone body

The level of plasma ketone body (β-hydroxybutyrate) was assessed by using a β-hydroxybutyrate assay kit (BioVision, Milpitas, CA), as described in the provided product manuals.

### Collection of cerebrospinal fluid

Cerebrospinal fluid (CSF) was collected from anesthetized mice after placing in the prone position and their cisterna magna were surgically exposed. The exposed meninges were penetrated to obtain CSF with a laboratory-produced tapered-tip capillary tube.

### Lactate measurement

Lactate levels were determined in hypothalamic tissues by high-performance liquid chromatography (HPLC). On the day of the experiment, tissues were homogenized with 150 μl lactate assay buffer (Abcam) and centrifuged at 4 °C at 10,000×*g* for 4 min. Supernatants were filtered through 10 kDa MW spin filters (Abcam) to remove all proteins. Lactate concentration was measured with an HPLC autosampler system (Shimadzu LC-20AT; Kyoto, Japan) equipped with four pumps and an SPD-M20A diode array detector. Then 20 μl of each sample was injected and the chromatographic separation was performed with an Inertsil C18 ODS-3 column (5 μm particle size, 4.6 mm × 250 mm; Japan). The analysis was isocratic at λmax: 210 nm, 65 °C (column temperature), and flow rate of 0.6 ml/min, with a mixture of water (adjusted to pH 2.0 with H_2_SO_4_) and acetonitrile as the mobile phase (95:5, v/v; total retention time was 30 min). The mobile phase was freshly prepared, passed through a 0.45 μm membrane filter to remove any particulate matter, and degassed by sonication before use. The sensitivity of the detector was set at 0.01 AUFS. Prior to sample injection, the column was equilibrated for at least 30 min with the mobile phase flowing through the system. Each sample was injected in triplicate with a relative standard deviation below 0.73% and 9.82% for standard samples and real samples, respectively. The lactate concentration was calculated against a standard curve using the area values of each pick.

### Cell culture treatment conditions

To ascertain the effect of high glucose (25 mM)-induced *Pdk2* transcription, we co-treated the cells with 1 µM of GSK0660 (a PPARβ/δ antagonist, Calbiochem) for 24 h. Similarly, to determine the effect of high glucose-induced altered glycolytic metabolism on inflammatory activation, we co-treated the cells with 5 mM of oxamate (a lactate dehydrogenase (LDH) inhibitor, Sigma-Aldrich) or 2 µM of GSK2837808A (an LDH inhibitor, Tocris Bioscience) for 24 hr. The embryonic mouse hypothalamic-N41cells (mHypoE-N41) expressing Npy/AgRP and embryonic mouse hypothalamic-N43/5 (mHypoE-N43/5) cells expressing POMC (Cellutions Biosystems Inc., kindly provided by Professor Eun-Kyoung Kim at Daegu Gyeongbuk Institute of Science & Technology, Republic of Korea) were maintained in low glucose DMEM with 10% fetal bovine serum (FBS) and 1% penicillin/streptomycin (Hyclone Laboratories Inc.) at 37 °C. To determine the role of lactic acid-induced AMP-activated protein kinase (AMPK) activity on neuropeptide transcription, we co-treated the cells with Compound C (an AMPK inhibitor; Calbiochem; 16 µM) or metformin (an AMPK activator; Sigma-Aldrich; 100 µM) for 24 h.

### Assessment of extracellular flux

An XF24 Extracellular Flux Analyzer (Seahorse Bioscience Inc., Billerica, MA) was used to determine the extracellular acidification rate (ECAR) and oxygen consumption rate (OCR). Briefly, astrocytes from WT and *Pdk2* KO were plated at a density of 50,000/well with a low-glucose (5.5 mM) DMEM medium in a Seahorse XF24 plate and cultured for 24 h, followed by replacement of the medium with new low- (5.5 mM) or high- (25 mM) glucose DMEM. One hour before the assay, the media were exchanged for XF24 media with 5.5 mM glucose. Rotenone or 2-Deoxy-d-glucose (2DG), carbonyl cyanide m-chlorophenyl hydrazine (CCCP), and oligomycin were diluted in XF24 media with glucose and loaded into the accompanying cartridge to achieve final concentrations of 2 μM, 1 μM, and 1 μg/ml with glucose concentrations of 5.5, 16, and 25 mM, respectively. Glucose and drug injections into the medium occurred at the time points specified before the analysis start point. Then, ECAR and OCR were monitored. Each cycle was set as: mix for 3 min, delay for 2 min, and measure for 3 min. Results were normalized to the total concentrations of protein; total protein was extracted from the cells immediately after the ECAR and OCR reading. Wave 2.6.0 software was used for the analysis of Seahorse data.

### Intracerebroventricular administration of PDK2-expressing recombinant adenovirus

The PDK2 recombinant adenovirus (Ad-PDK2-GFP) construct was generated with a pAd-Track-CMV shuttle vector^66^. Before 7 days of STZ administration in mice, Ad-PDK2-GFP or Ad-GFP (1.0 × 10^9^ plaque-forming units; pfu) was delivered into the third ventricle by intracerebroventricular (icv) injection at the midline coordinate of 0.5 mm posterior to the bregma and 5 mm below the surface of the skull. On day 7 after adenovirus infection, STZ was administered intraperitoneally (ip), and food intake, and blood glucose were assessed up to 3 weeks after diabetes induction. On day 28 after adenovirus infection, the mice were sacrificed, and hypothalamic tissues were collected rapidly.

### Intracerebroventricular cannulation and injection of pharmacological inhibitors of hypothalamic inflammation, PDK2, and lactic acid production

Mice were anesthetized and implanted with a guide cannula (Plastics One) into the third ventricle at the midline coordinates of 0.5 mm posterior to the bregma and 5 mm below the surface of the skull. After at least 1 week of recovery from surgery, 3 μl vehicle (artificial cerebrospinal fluid, aCSF) with or without inhibitors was injected through the cannula into the third ventricle. To determine the role of diabetes-induced hypothalamic inflammation, PDK2, and lactic acid production in feeding behavior and related pathologies, Bay 11-7085 (an inhibitor of I-κB phosphorylation, Santa Cruz Biotechnology, 1 µg), Ki20227 (an inhibitor of CSF1R, Tocris Bioscience, 1 µg), 4-[3-chloro-4-[[(2 R)-3,3,3-trifluoro-2-hydroxy-2-methylpropanoyl]amino]phenyl]sulfonyl-N,N-dimethylbenzamide (AZD7545; a PDK2 inhibitor; 6.4 nM of CSF concentration), and 3-[[3-[(Cyclopropylamino)sulfonyl]-7-(2,4-dimethoxy-5-pyrimidinyl)-4-quinolinyl]amino]-5-(3,5-difluorophenoxy) benzoic acid (GSK2837808A, a small-molecule inhibitor of LDH; 2 µM of CSF concentration) or sodium oxamate (Oxamate, 25 µg) or vehicle (aCSF or 1% (v/v) DMSO) solution were delivered by multiple icv injections to mice. Daily 12-h food intake in the dark phase and body weight were measured at the time of injections.

### Electrophysiology

WT mice (12–17 days old) were decapitated under ketamine anesthesia (50 mg/kg, ip). The brains were quickly removed and immersed in an ice-cold Ringer solution composed of (in mM): 119 NaCl, 2.5 KCl, 1.3 MgSO_4_, 2.5 CaCl_2_, 1.0 NaH_2_PO_4_, 26.2 NaHCO_3_, and 11 glucose that had been saturated with 95% O_2_ and 5% CO_2_. The brains were dissected and transverse slices (400 μm thick) were prepared with a microslicer (VT1000S; Leica, Nussloch, Germany). Slices containing the hypothalamus were placed in a humidified holding chamber for at least 1 h before mechanical dissociation. For the dissociation, slices were transferred into a 35-mm culture dish (Primaria 3801; Becton Dickinson, Rutherford, NJ) that contained a standard external solution (see below), and the ARC region was identified under a binocular microscope (SMZ-1; Nikon, Tokyo, Japan)^67^. Briefly, mechanical dissociation was accomplished with a custom-built vibration device and a fire-polished glass pipette oscillating at ~50–60 Hz (0.3–0.5 mm) on the surface of the ARC region. Slices were removed and mechanically dissociated neurons were allowed to settle and adhere to the bottom of the dish for 15 min.

All electrophysiological recordings were performed using the whole-cell patch-clamp technique with an Axopatch 200B amplifier (Molecular Devices, Union City, CA). Neurons were clamped at a holding potential of −40 mV. Patch pipettes were made from borosilicate capillary glass (1.5 mm outer diameter, 0.9 mm inner diameter; G-1.5; Narishige, Tokyo, Japan) with a pipette puller (P-97; Sutter Instrument Co., Novato, CA). The resistance of the recording pipettes filled with the internal solution was 3–4 MΩ. The liquid junction potential and the pipette capacitance were compensated for. Neurons were viewed under phase contrast or fluorescence on an inverted microscope (TE-2000; Nikon). Membrane currents were filtered at 1 kHz (Axopatch 200B) and digitized at 4 kHz (Digidata 1440; Molecular Devices); the results were stored in a computer database with pCLAMP 10.6 (Molecular Devices). When recording, we periodically delivered 10 mV hyperpolarizing step pulses (30 ms in duration) to monitor the access resistance. All experiments were performed at room temperature (22–24 °C). The internal solution used to record the NMDA-induced currents (I_NMDA_) was composed of (in mM): 140 Cs-methanesulfonate, 10 CsCl, 2 EGTA, 2 Mg-ATP, and 10 HEPES (pH 7.2 with Tris-base). The internal solution used to record spontaneous synaptic currents and action potentials was composed of (in mM) 140 K-methanesulfonate, 10 KCl, 2 EGTA, 2 Mg-ATP, and 10 HEPES (pH 7.2 with Tris-base). The external solution was composed of (in mM) 150 NaCl, 3 KCl, 2 CaCl_2_, 1 MgCl_2_, 10 HEPES, 10 glucose, 0.001 strychnine, and 10 glycine (pH 7.4 with Tris-base). sEPSC_NMDA_ were recorded in the Mg^2+^-free external solution. l-lactate or D-lactate (7.1 mM) was replaced with equimolar NaCl. The I_NMDA_ amplitudes were calculated by subtracting the baseline from the peak amplitude. sEPSC_NMDA_ were counted and analyzed using a threshold search method with an amplitude threshold of 15 pA (pCLAMP 10.6). The average values of the frequency and amplitude of all sEPSC_NMDA_ during the control period (5–10 min) were calculated for each recording, and the frequency and amplitude of all the events during the application of l-lactate (5 min) were normalized to these values. The effects of l-lactate were quantified as a percentage change in the frequency and amplitude of sEPSC_NMDA_ compared to the respective control values. The inter-event intervals and amplitudes of a large number of synaptic events obtained from the same neuron were examined by constructing cumulative probability distributions, which were then compared using the Kolmogorov–Smirnov (K–S) test with SPSS program version 26.0 K (SPSS Inc., Chicago, IL).

### Single-cell RT-PCR

After whole-cell patch-clamp recording, the contents of the patched neuron, including mRNA, were aspirated by applying a gentle suction through the recording pipette. Then, the harvested material in the patch pipette was expelled into a PCR tube, and reverse transcription and first PCR reactions were performed in the same tube with a one-step RT-PCR kit (Qiagen, Hilden, Germany). Primers used for RT-PCR were as follows: *Npy*; (1st) forward, 5′-CTT CTC TCA CAG AGG CAC CCA-3′, reverse, 5′-GGG ACA GGC AGA CTG GTT TCA-3’, (2nd) forward, 5′-TGG ACT GAC CCT CGC TCT ATC-3′, reverse, 5′-CCC ATC ACC ACA TGG AAG GGT-3′ (product size 269 bp), *Pomc*; (1st) forward, 5′- CTG ACA CGT GGA AGA TGC CGA-3′, reverse, 5′-CC ACT CGT TCT CAG CAA CGT-3′, (2nd) forward, 5′-ACC ACG GAG AGC AAC CTG CT-3′, reverse, 5’-CAT GGA GTA GGA GCG CTT GC-3′ (product size 270 bp). The nested second PCR was performed using GoTaq^®^ DNA polymerase (Promega) and each first-round PCR product was used as a template (2 µl). Amplified PCR products were electrophoresed in 2% agarose gels, to which RedSafe^TM^ Nucleic Acid Staining Solution had been added, and the gels subsequently photographed.

### Astrocyte or neuron-specific *Pdk2* knockout

To ablate *Pdk2* in hypothalamic astrocytes or neurons, we used *Pdk2* floxed mice and adeno-associated virus (AAV) particles (serotype 5 or 2) expressing eYFP (Viral vector facility, Neuroscience Center Zurich, University of Zurich and ETH Zurich) or mCherry/mCherry-Cre (University North Carolina Vector Core) under control of the GFAP or human synapsin 1 (hSyn) promoters targeting astrocytes or neurons, respectively. The *Pdk2* floxed mice were kindly provided by Professor In-Kyu Lee at Kyungpook National University. We stereotactically injected AAV5-GFAP-eYFP or AAV2-hSyn-mCherry or AAV5-GFAP-mCherry-Cre or AAV2-hSyn-mCherry-Cre particles bilaterally (4.3 × 10^9^ virus particles per side) into the mediobasal hypothalamus (MBH) of *Pdk2* floxed mice. Stereotaxic coordinates were 1.5 mm posterior and 0.3 mm lateral to the bregma, and 5.8 mm ventral from the dura. Astrocyte or neuron-specific knockout of *Pdk2* was confirmed by isolating fluorescence-labeled cells from the hypothalamus using fluorescence-activated cell sorting (FACS). For this, hypothalamic tissues were incubated with a solution of 1x Accutase (ThermoFisher) supplemented with 80 U/ml DNase I (Sigma) at 37 °C for 20 min, followed by gentle trituration in Hybernate A media (Invitrogen) along with 1% FBS. The cell suspension was prepared by passing it through a 70 μm filter, overlaying it on top of an isotonic Percoll gradient (top phase: 11%, 31 ml; bottom phase: 30%, 2 ml), and centrifuging it at 400 × *g* for 5 min at 4 °C. Dissociated mixed cells were retrieved using a pipette carefully from the interface between 11% and 13% phases and pelleted by centrifugation. The cell pellet was washed twice and resuspended in 500 μl 1x PBS + 3% FBS and then subjected to FACS using an automated high-speed cell sorter (Beckman Coulter, MoFlo XDP) and Summit software (version 5.2) for analysis. mCherry- or eYFP-positive cells were sorted and used for total RNA extraction and gene expression analysis.

### Quantification and statistical analysis

For the analysis of immunohistochemistry or Western blot band intensities, the whole image area or each band was marked, and the mean intensity or number of particles were measured with Fiji (ImageJ, version 1.47) software (National Institutes of Health, Bethesda, MD). The background intensity of each band was measured and deducted from the target band values. Pearson’s correlation coefficient method was used with the help of image J software for the quantification of PDK2 cellular co-localization because this method gives an objective quantification of the extent of overlap of fluorescence at a pixel-by-pixel basis for two different fluorophores imaged from two different channels. For the immunohistochemical analysis, five to six microscopic images were chosen randomly for statistical analysis. The sample size for each experiment was determined by power analysis using G Power software (version 3.1). The statistical power was calculated before data collection based on information from previous studies to decide the sample size needed and then adjusted in cases when the result turned out to be non-significant. The power of the study was considered to be minimum 0.8. Experiments were repeated at least two to three times with similar results. The inter-assay coefficient of variation was <20%, which is considered to indicate confidence in the results. Statistical analysis was performed with either a Student’s *t*-test or a one/two-way ANOVA and Tukey’s post hoc test with GraphPad Prism (version 8). Probability value differences <0.05, 0.01, or 0.001 (*p* < 0.05, 0.01, or 0.001) were considered statistically significant. All results are presented as the mean ± S.E.M.

### Reporting summary

Further information on research design is available in the Nature Research Reporting Summary linked to this article.

## Supplementary information

Supplementary Information

Reporting Summary

## Data Availability

The authors declare that all data supporting the findings of this study are available within the paper and its supplementary information files. Source data are provided with this paper.

## Source data

Source Data

## References

[1] Laye S (2000). Endogenous brain IL-1 mediates LPS-induced anorexia and hypothalamic cytokine expression. Am. J. Physiol. Regul. Integr. Comp. Physiol..

[2] Laviano A (1999). Peripherally injected IL-1 induces anorexia and increases brain tryptophan concentrations. Adv. Exp. Med. Biol..

[3] Zhang X (2008). Hypothalamic IKKbeta/NF-kappaB and ER stress link overnutrition to energy imbalance and obesity. Cell.

[4] Kreutzer C (2017). Hypothalamic inflammation in human obesity is mediated by environmental and genetic factors. Diabetes.

[5] Thaler JP (2012). Obesity is associated with hypothalamic injury in rodents and humans. J. Clin. Invest..

[6] Milanski M (2012). Inhibition of hypothalamic inflammation reverses diet-induced insulin resistance in the liver. Diabetes.

[7] Zhang Y, Reichel JM, Han C, Zuniga-Hertz JP, Cai D (2017). Astrocytic process plasticity and IKKbeta/NF-kappaB in central control of blood glucose, blood pressure, and body weight. Cell Metab..

[8] Cai D, Khor S (2019). “Hypothalamic microinflammation” paradigm in aging and metabolic diseases. Cell Metab..

[9] Kleinridders A (2009). MyD88 signaling in the CNS is required for development of fatty acid-induced leptin resistance and diet-induced obesity. Cell Metab..

[10] Mendes NF (2018). TGF-beta1 down-regulation in the mediobasal hypothalamus attenuates hypothalamic inflammation and protects against diet-induced obesity. Metabolism.

[11] Purkayastha S, Zhang G, Cai D (2011). Uncoupling the mechanisms of obesity and hypertension by targeting hypothalamic IKK-beta and NF-kappaB. Nat. Med..

[12] Dorfman MD (2017). Sex differences in microglial CX3CR1 signalling determine obesity susceptibility in mice. Nat. Commun..

[13] Purkayastha S (2011). Neural dysregulation of peripheral insulin action and blood pressure by brain endoplasmic reticulum stress. Proc. Natl Acad. Sci. USA.

[14] Meng Q, Cai D (2011). Defective hypothalamic autophagy directs the central pathogenesis of obesity via the IkappaB kinase beta (IKKbeta)/NF-kappaB pathway. J. Biol. Chem..

[15] Valdearcos M (2014). Microglia dictate the impact of saturated fat consumption on hypothalamic inflammation and neuronal function. Cell Rep..

[16] Douglass JD, Dorfman MD, Fasnacht R, Shaffer LD, Thaler JP (2017). Astrocyte IKKbeta/NF-kappaB signaling is required for diet-induced obesity and hypothalamic inflammation. Mol. Metab..

[17] Lee CH (2018). Hypothalamic macrophage inducible nitric oxide synthase mediates obesity-associated hypothalamic inflammation. Cell Rep..

[18] Morari J (2014). Fractalkine (CX3CL1) is involved in the early activation of hypothalamic inflammation in experimental obesity. Diabetes.

[19] Rahman MH, Bhusal A, Lee WH, Lee IK, Suk K (2018). Hypothalamic inflammation and malfunctioning glia in the pathophysiology of obesity and diabetes: translational significance. Biochem. Pharmacol..

[20] Andre C (2017). Inhibiting microglia expansion prevents diet-induced hypothalamic and peripheral inflammation. Diabetes.

[21] Valdearcos M (2017). Microglial inflammatory signaling orchestrates the hypothalamic immune response to dietary excess and mediates obesity susceptibility. Cell Metab..

[22] Chen N (2016). Direct modulation of GFAP-expressing glia in the arcuate nucleus bi-directionally regulates feeding. Elife.

[23] Hu P (2015). Loss of survival factors and activation of inflammatory cascades in brain sympathetic centers in type 1 diabetic mice. Am. J. Physiol. Endocrinol. Metab..

[24] Hu P (2013). CNS inflammation and bone marrow neuropathy in type 1 diabetes. Am. J. Pathol..

[25] Daneman D (2006). Type 1 diabetes. Lancet.

[26] Cabrera SM, Henschel AM, Hessner MJ (2016). Innate inflammation in type 1 diabetes. Transl. Res..

[27] Sollier M (2019). Diabetes mellitus, extreme insulin resistance, and hypothalamic-pituitary langerhans cells histiocytosis. Case Rep. Endocrinol..

[28] Nair S (2019). Lipopolysaccharide-induced alteration of mitochondrial morphology induces a metabolic shift in microglia modulating the inflammatory response in vitro and in vivo. Glia.

[29] Jiang T, Cadenas E (2014). Astrocytic metabolic and inflammatory changes as a function of age. Aging Cell.

[30] Motori E (2013). Inflammation-induced alteration of astrocyte mitochondrial dynamics requires autophagy for mitochondrial network maintenance. Cell Metab..

[31] Kim JD, Yoon NA, Jin S, Diano S (2019). Microglial UCP2 mediates inflammation and obesity induced by high-fat feeding. Cell Metab..

[32] Patel MS, Roche TE (1990). Molecular biology and biochemistry of pyruvate dehydrogenase complexes. FASEB J..

[33] Rogatzki MJ, Ferguson BS, Goodwin ML, Gladden LB (2015). Lactate is always the end product of glycolysis. Front. Neurosci..

[34] Rahman MH (2016). Pyruvate dehydrogenase kinase-mediated glycolytic metabolic shift in the dorsal root ganglion drives painful diabetic neuropathy. J. Biol. Chem..

[35] Jha MK (2015). Metabolic connection of inflammatory pain: pivotal role of a pyruvate dehydrogenase kinase-pyruvate dehydrogenase-lactic acid axis. J. Neurosci..

[36] Xing G, Ren M, O’Neill JT, Verma A, Watson WD (2012). Controlled cortical impact injury and craniotomy result in divergent alterations of pyruvate metabolizing enzymes in rat brain. Exp. Neurol..

[37] Varela L, Horvath TL (2012). Leptin and insulin pathways in POMC and AgRP neurons that modulate energy balance and glucose homeostasis. EMBO Rep..

[38] Iwata K (2011). Involvement of brain ketone bodies and the noradrenergic pathway in diabetic hyperphagia in rats. J. Physiol. Sci..

[39] Degenhardt T (2007). Three members of the human pyruvate dehydrogenase kinase gene family are direct targets of the peroxisome proliferator-activated receptor beta/delta. J. Mol. Biol..

[40] Minokoshi Y (2004). AMP-kinase regulates food intake by responding to hormonal and nutrient signals in the hypothalamus. Nature.

[41] Namkoong C (2005). Enhanced hypothalamic AMP-activated protein kinase activity contributes to hyperphagia in diabetic rats. Diabetes.

[42] Yang Y (2017). Exendin-4 reduces food intake via the PI3K/AKT signaling pathway in the hypothalamus. Sci. Rep..

[43] Solon CS (2012). Taurine enhances the anorexigenic effects of insulin in the hypothalamus of rats. Amino Acids.

[44] Tang F (2014). Lactate-mediated glia-neuronal signalling in the mammalian brain. Nat. Commun..

[45] Yang J (2014). Lactate promotes plasticity gene expression by potentiating NMDA signaling in neurons. Proc. Natl Acad. Sci. USA.

[46] Valdearcos M, Xu AW, Koliwad SK (2015). Hypothalamic inflammation in the control of metabolic function. Annu. Rev. Physiol..

[47] Cai D (2013). Neuroinflammation and neurodegeneration in overnutrition-induced diseases. Trends Endocrinol. Metab..

[48] Walz W, Mukerji S (1988). Lactate release from cultured astrocytes and neurons: a comparison. Glia.

[49] Halim ND (2010). Phosphorylation status of pyruvate dehydrogenase distinguishes metabolic phenotypes of cultured rat brain astrocytes and neurons. Glia.

[50] Magistretti PJ, Allaman I (2018). Lactate in the brain: from metabolic end-product to signalling molecule. Nat. Rev. Neurosci..

[51] Itoh Y (2003). Dichloroacetate effects on glucose and lactate oxidation by neurons and astroglia in vitro and on glucose utilization by brain in vivo. Proc. Natl Acad. Sci. USA.

[52] Diaz-Garcia CM (2017). Neuronal stimulation triggers neuronal glycolysis and not lactate uptake. Cell Metab..

[53] Lizarbe B, Soares AF, Larsson S, Duarte JMN (2018). Neurochemical modifications in the hippocampus, cortex and hypothalamus of mice exposed to long-term high-fat diet. Front. Neurosci..

[54] Krzymien J, Karnafel W (2013). Lactic acidosis in patients with diabetes. Pol. Arch. Med. Wewn..

[55] Andersson AK, Ronnback L, Hansson E (2005). Lactate induces tumour necrosis factor-alpha, interleukin-6 and interleukin-1beta release in microglial- and astroglial-enriched primary cultures. J. Neurochem..

[56] Kim J (2018). The involvement of 4-1BB/4-1BBL signaling in glial cell-mediated hypothalamic inflammation in obesity. FEBS Open Bio.

[57] Rahman MH, Kim MS, Lee IK, Yu R, Suk K (2018). Interglial crosstalk in obesity-induced hypothalamic inflammation. Front. Neurosci..

[58] Covelo A, Araque A (2018). Neuronal activity determines distinct gliotransmitter release from a single astrocyte. Elife.

[59] Long YC, Zierath JR (2006). AMP-activated protein kinase signaling in metabolic regulation. J. Clin. Invest..

[60] Cha SH, Lane MD (2009). Central lactate metabolism suppresses food intake via the hypothalamic AMP kinase/malonyl-CoA signaling pathway. Biochem. Biophys. Res. Commun..

[61] Jiang P (2013). Adenosine monophosphate-activated protein kinase overactivation leads to accumulation of alpha-synuclein oligomers and decrease of neurites. Neurobiol. Aging.

[62] Parsons MP, Hirasawa M (2010). ATP-sensitive potassium channel-mediated lactate effect on orexin neurons: implications for brain energetics during arousal. J. Neurosci..

[63] Rau AR, Hentges ST (2017). The relevance of AgRP neuron-derived GABA inputs to POMC neurons differs for spontaneous and evoked release. J. Neurosci..

[64] Essner RA (2017). AgRP neurons can increase food intake during conditions of appetite suppression and inhibit anorexigenic parabrachial neurons. J. Neurosci..

[65] Jeoung NH, Rahimi Y, Wu P, Lee WN, Harris RA (2012). Fasting induces ketoacidosis and hypothermia in PDHK2/PDHK4-double-knockout mice. Biochem. J..

[66] He TC (1998). A simplified system for generating recombinant adenoviruses. Proc. Natl Acad. Sci. USA.

[67] Akaike N, Moorhouse AJ (2003). Techniques: applications of the nerve-bouton preparation in neuropharmacology. Trends Pharmacol. Sci..

