# Supplementary material for: Astrocytic pyruvate dehydrogenase kinase-2 is involved in hypothalamic inflammation in mouse models of diabetes
Source: Nat Commun. 2020 Nov 20;11:5906. doi: 10.1038/s41467-020-19576-1 (PMC7680139; doi:10.1038/s41467-020-19576-1)
Supplement: Supplementary file 1 — Supplementary Information [file 41467_2020_19576_MOESM1_ESM.pdf]

Supplementary Fig. 1

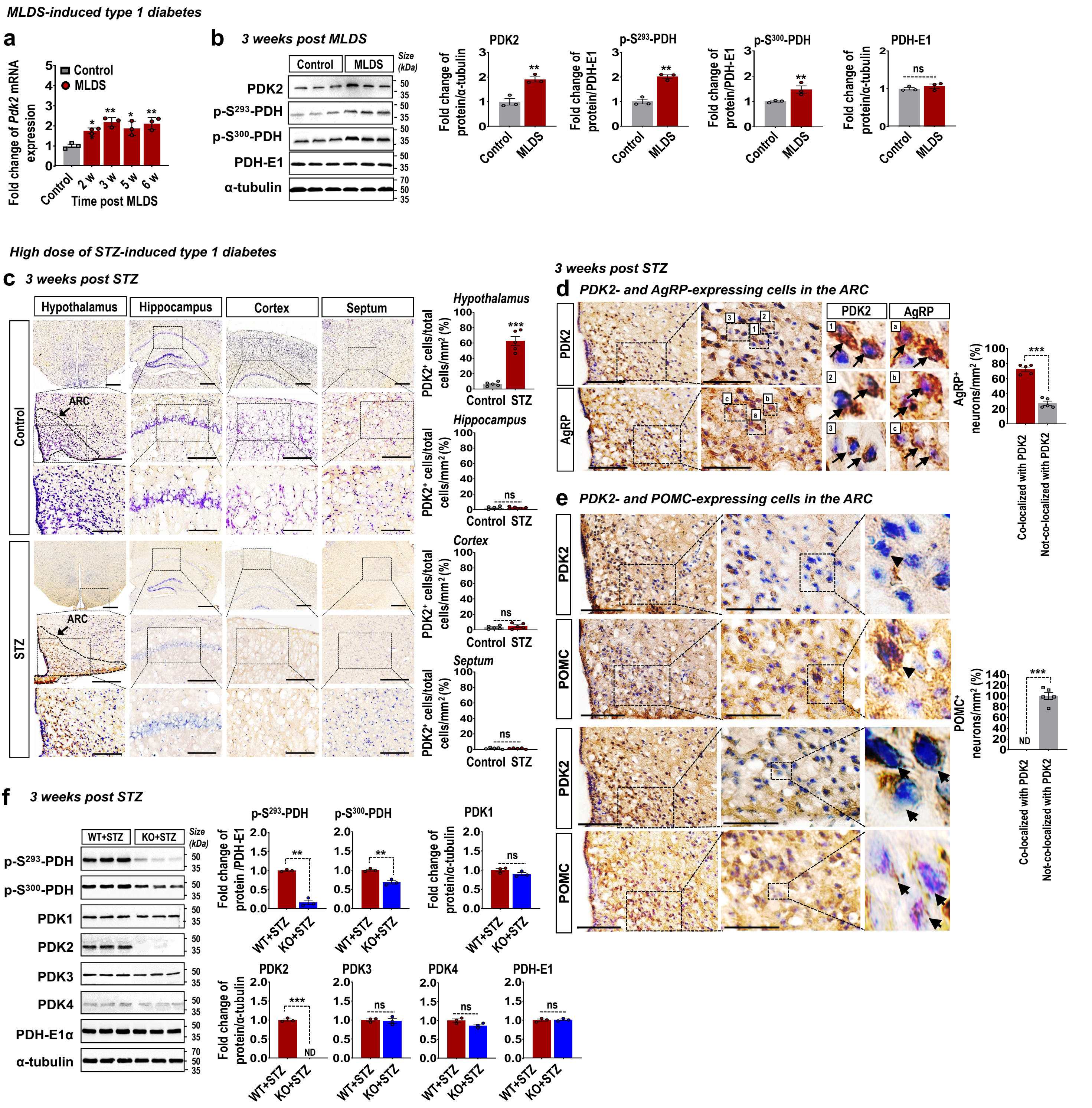

Supplementary Fig. 1 ***Expression of PDK and phosphorylated-PDH in diabetic brains.*** The expression of *Pdk2* mRNA (*p= 0.0256, 2 w; **p= 0.0012, 3 w; *p= 0.0109, 5 w; and **p= 0.0020, 6 w) in the hypothalamus at 2, 3, 5, and 6 w following MLDS injection (a) was assessed by real-time RT-PCR. PDK2 (**p= 0.0059, MLDS), phosphorylated-PDH (p-S^{293}-PDH: **p= 0.0012, MLDS; and p-S^{300}-PDH: **p= 0.0030, MLDS), and PDH-E1 protein levels in the hypothalamus at 3 w post-MLDS injection (b) were assessed by Western blot analysis. Quantification of the band intensities is shown in the adjacent graphs. Phosphorylated-PDH band quantification was

based on normalization against PDH-E1. DAB staining of mouse brain tissues shows PDK2 immunoreactivity in the hypothalamus (***p= 1.7E-5, STZ), hippocampus, cortex, and septum at 3 w post-vehicle/STZ injection (c). Arrows indicate PDK2 immunoreactivity in the hypothalamic ARC. Scale bars indicate 400 μm (upper row), 200 μm (middle row), and 100 μm (lower row). DAB staining of brain serial sections shows the localization of PDK2 as well as (d) AgRP (***p= 3.1E-6, co-localized with PDK2) and (e) POMC (***p= 4.1E-7, not co-localized with PDK2)-positive cells in the hypothalamic ARC at 3 w post-vehicle/STZ injection. Long arrows indicate both PDK2 and AGRP-positive neurons (d). Arrowheads indicate the PDK2-negative and POMC-positive neurons (e). Short arrows indicate the PDK2-positive and POMC-negative neurons (e). Quantification of the percentage of PDK2-positive or -negative cells per mm^2 is shown in the adjacent images. Microscope data were assembled using five randomly selected fields captured at same magnification. Scale bars indicate 200 μm (left image) and 100 μm (middle image). To determine whether PDK2 is a major regulator of PDH phosphorylation in the diabetic hypothalamus, we assessed PDK1- 4 (***p= 2.9E-6, KO+STZ for PDK2), phosphorylated-PDH (p-S^{293}-PDH: **p= 0.0020, KO+STZ; and p-S^{300}-PDH: **p= 0.0031, KO+STZ), and PDH-E1 protein levels at 3 w post-vehicle/STZ injection using Western blot analysis (f). Western blot band quantification for p-PDH was based on normalization against PDH-E1, and PDK2 and PDH-E1 were normalized to α-tubulin. *$p < 0.05$, **$p < 0.01$, or ***$p < 0.001$ versus the vehicle-treated control animals. One-way ANOVA with Tukey's post-hoc test (a), two-tailed Student's *t*-test (b-f), n = 3 (a-b, f), and n=5 (c-e); mean ± SEM. Source data are provided as a Source Data file. w, week(s); MLDS, multiple low doses of streptozotocin; DAB, 3, 3′-Diaminobenzidine; ARC, arcuate nucleus; ns, not significant; ND, not detected.

Supplementary Fig. 2

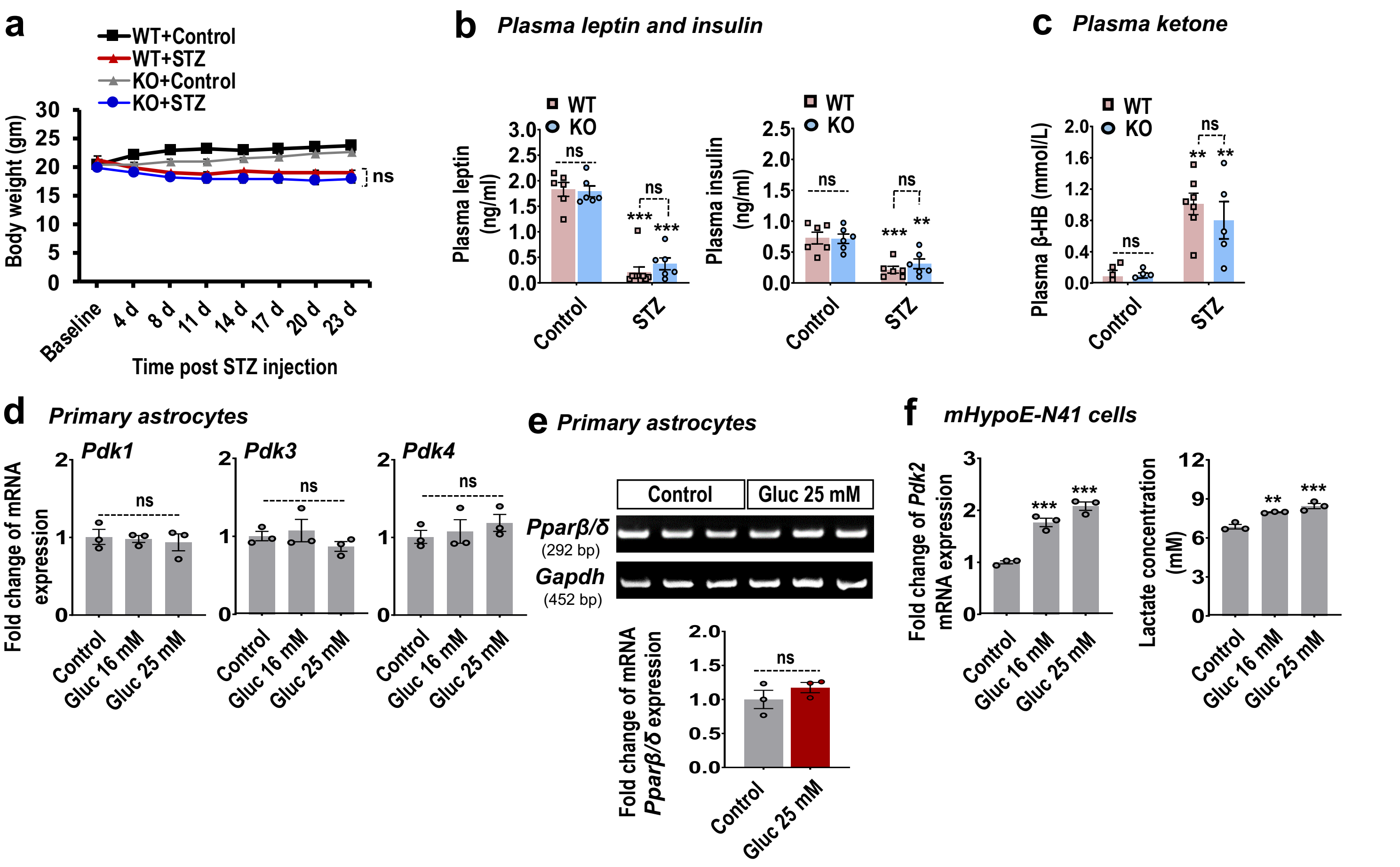

Supplementary Fig. 2 ***Effect of Pdk2 deficiency on body weight, plasma leptin, insulin, and ketone body levels: High-glucose-related changes in Pdk1, 3, and 4, and Pparβ/δ mRNA expression in primary astrocytes and Pdk2 mRNA expression in mHypoE-N41 cells and lactate level in such culture media.*** Body weight (a) of WT and *Pdk2* KO mice were assessed following vehicle/STZ administration. (b) Leptin (p= 0.7296, KO+STZ), insulin (p= 0.8229, KO+STZ), and (c) β-hydroxybutyrate (β-HB: p= 0.7197, KO+STZ) levels in the plasma at 3 w post-vehicle/STZ injection. Leptin and insulin levels were measured by ELISA. The relative expression of *Pdk1*, *3*, and *4* (d), and *Pparβ/δ* (e) mRNA in cultured astrocytes (maintained with low glucose, 5.5 mM) was assessed after high-glucose (16 mM or 25 mM) treatment for 24 hr by real-time (for *Pdks*) and conventional (*Pparβ/δ*) RT-PCR. The relative expression of *Pdk2* mRNA (***p= 0.0005, Gluc 16 mM; and ***p= 7.3E-5 Gluc 25 mM) in mHypoE-N41 cells (maintained with low glucose, 5.5 mM) was assessed after high-glucose (16 mM and 25 mM) treatment for 24 hr by real-time RT-PCR (f). Extracellular lactate (**p= 0.0052, Gluc 16 mM; ***p= 0.0008, Gluc 25 mM) was assessed by HPLC analysis after mHypoE-N41 cells were exposed to high glucose (16 mM and 25 mM) for 24 hr (f). **p < 0.01, or ***p < 0.001 versus control animals or non-treated group. Two-way ANOVA (a-c) and one-way ANOVA (d, f) with Tukey's post-hoc test, two-tailed Student's *t*-test (e), n = 5 for control and 6 for STZ groups (a), n = 9 for WT+STZ (plasma leptin) and 6 for other groups (b), n = 7 for WT+STZ and 5 for other groups (c), and n = 3 (d-f); mean ± SEM. Value of 'n' for a-c indicates the number of animal. Source data are provided as a Source Data file. WT, wild-type; KO, knockout; STZ, streptozotocin; Gluc, glucose; w, week(s); intracerebroventricular, icv; bp, base pair; ns, not significant.

Supplementary Fig. 3

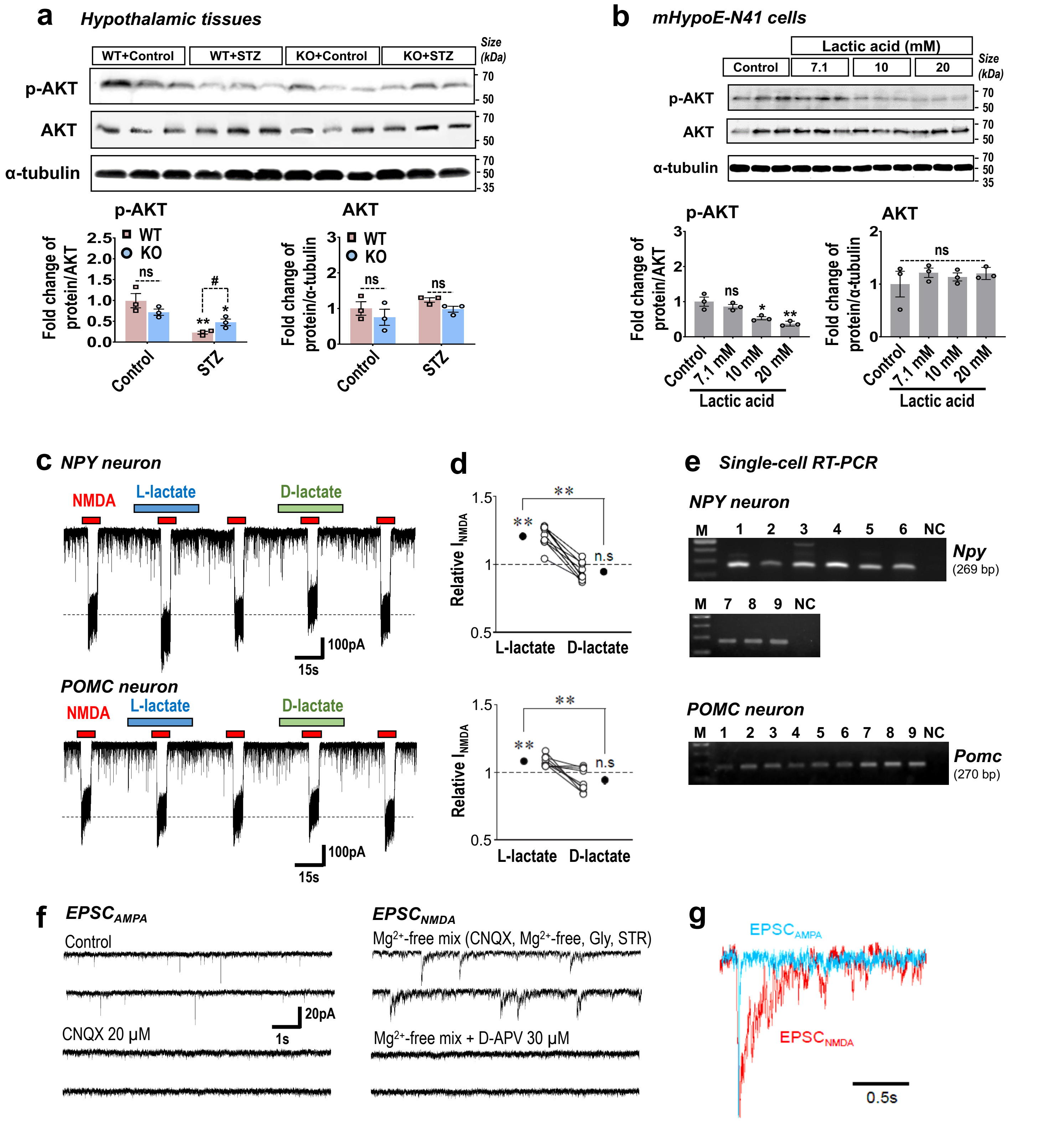

Supplementary Fig. 3 ***The role of PDK2 and lactic acid in regulating the expression of phosphorylated-AKT and activity of food intake-related neurons in the diabetic mouse hypothalamus.*** p-AKT (#p= 0.0382, KO+STZ) and AKT protein levels in hypothalamic tissues isolated from WT and *Pdk2* KO mice at 3 w post-STZ/vehicle injection were assessed by Western blot analysis (a). mHypoE-N41 cells were treated with lactic acid (7.1, 10, and 20 mM)

for 30 min (b). The expression of p-AKT (p= 0.5890, 7.1 mM; *p= 0.0113, 10 mM; and **p= 0.0020, 20 mM) and AKT proteins was assessed by Western blot analysis. Quantification of Western blot band intensities is shown in the adjacent graphs. Western blot quantification for p-AKT was based on normalization against AKT, and AKT was normalized to α-tubulin. Representative traces of I_{NMDA} induced by NMDA (100 μM) in the presence or absence of L-lactate (7.1 mM) and D-lactate (7.1 mM) in NPY/AgRP-expressing neurons (upper) and POMC-expressing neurons (lower) (c). L-Lactate-induced changes of the NMDA-induced currents (**p= 0.0012, D-lactate for NPY or POMC neuron) in NPY/AgRP-expressing and POMC-expressing neurons (d). Open and closed circles represent the value from individual neurons and their average, respectively. Single-cell RT-PCR analysis from patched neurons. The transcripts for NPY (269 bp) and POMC (270 bp) were simultaneously detected from nine individual neurons tested, respectively (e). Representative traces of spontaneous excitatory postsynaptic currents recorded at a holding potential of −60 mV (f). The sEPSCs-mediated AMPA receptors ($sEPSC_{AMPA}$) were completely eliminated by adding 20 μM CNQX, a selective AMPA/KA receptor antagonist (left panel). In these conditions, exposure of Mg^{2+}-free mixture (20 μM CNQX, 1 μM strychnine, 10 μM glycine, and Mg^{2+}-free) to the same neurons reappeared sEPSCs mediated NMDA receptors ($sEPSC_{NMDA}$), which were completely blocked by adding 30 μM D-APV, a selective NMDA receptor antagonist (right panel). Kinetic of single $sEPSC_{AMPA}$ and $sEPSC_{NMDA}$ (g). Decay time constants were 3.9 ± 0.3 ms for $sEPSC_{AMPA}$ (n = 7) and 218.4 ± 18.6 ms $sEPSC_{NMDA}$ (n = 7). *p < 0.05 or **p < 0.01 versus controls; #p < 0.05 versus indicated groups. Two-way ANOVA (a), one-way ANOVA with Tukey's post-hoc test (b), two-tailed Paired *t*-test (d), n=3 independent animals (a) or sister wells (b), n=9 individual neurons (c-e), and n = 7 (g); mean ± SEM. Source data are provided as a Source Data file. w, week(s); ns, not significant.

Supplementary Fig. 4

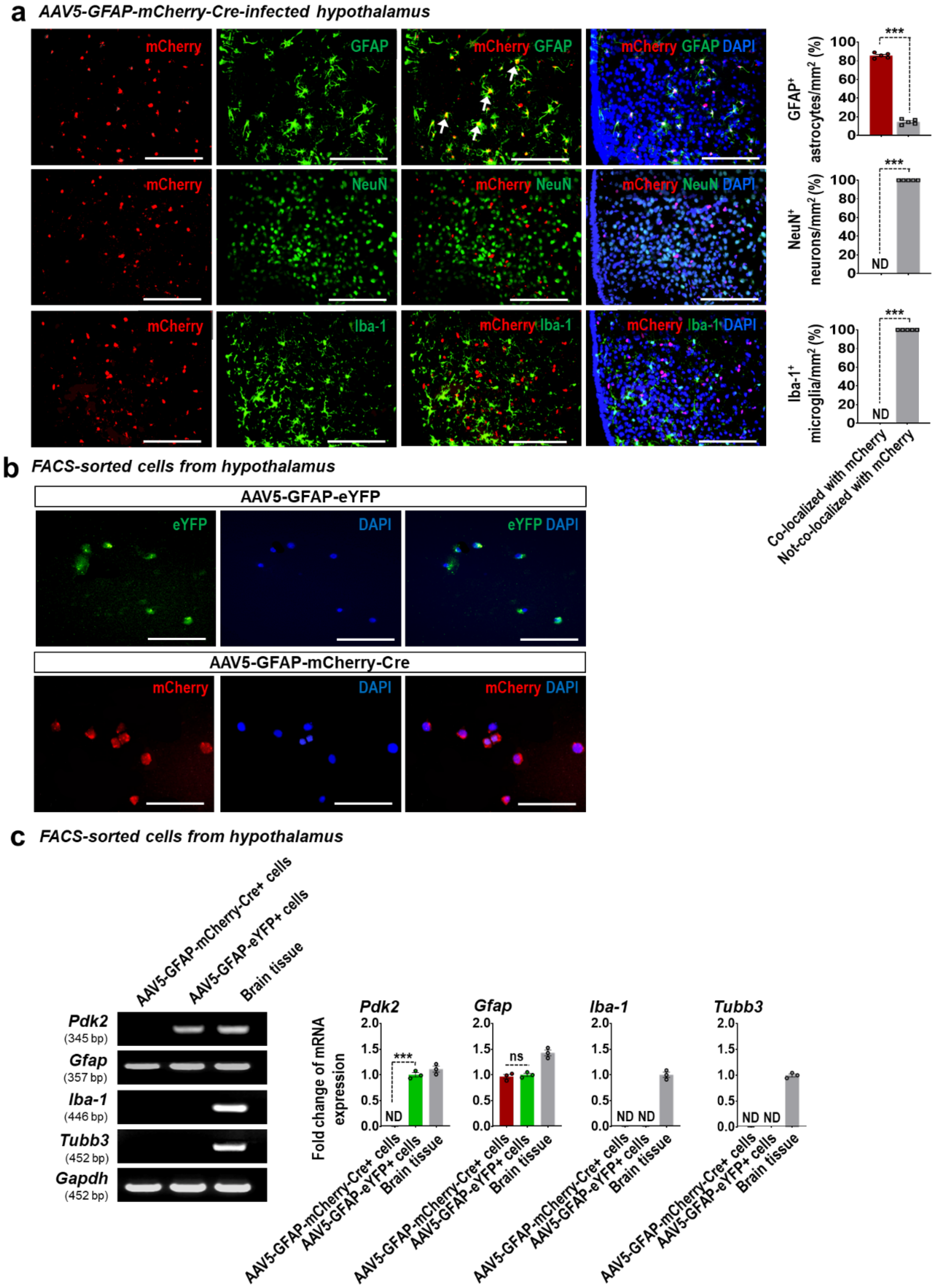

Supplementary Fig. 4 ***Validation of AAV5-GFAP-mCherry-Cre-mediated Pdk2 recombination in hypothalamic astrocytes.*** To ablate *Pdk2* in hypothalamic astrocytes, we administered AAV5-GFAP-eYFP or AAV5-GFAP-mCherry-Cre particles (4.3×10^9 virus particles per side) stereotatically into the bilateral mediobasal hypothalamus of *Pdk2* floxed mice. GFAP, NeuN, and Iba-1 immunofluorescence staining of brain tissues isolated from virus-injected *Pdk2* floxed mice confirmed an efficient virus infection specific for astrocytes after 4 w of virus

administration (a). Quantification of the percentage of mCherry-positive or -negative GFAP-expressing astrocytes (***p= 1.4E-10, co-localized with mCherry), NeuN-expressing neurons (***p= 1E-15, not co-localized with mCherry), or Iba-1-expressing microglia (***p= 1.4E-15, not co-localized with mCherry) per mm^2 is shown within the adjacent graphs. FACS-sorted mCherry- and eYFP-positive hypothalamic cells were confirmed under fluorescent microscopy and used for RT-PCR analysis (b). Experiments were repeated at least two times and the qualitative results were similar. The expression of *Pdk2* (***p= 5.7E-6, AAV5-GFAP-mCherry-Cre^+ cells), *Gfap*, *Iba-1*, and *Tubb3* mRNA in FACS-sorted mCherry- and eYFP-positive cells confirmed successful *Pdk2* recombination in hypothalamic astrocytes *in vivo* (c). Microscope data were accumulated using five randomly selected fields captured at same magnification. mRNA expression profiles are displayed as the fold increase of gene expression normalized to *Gapdh*. The fold change was made for *Pdk2* and *Gfap* against AAV5-GFAP-eYFP-infected cells and *Iba-1* and *Tubb3* against brain tissue. Scale bars indicate 200 □m. ***$p < 0.001$ versus indicated groups. Two-tailed Student's *t*-test (a), one-way ANOVA with Tukey's post-hoc test (c), n = 5 independent animals (a), n=2 independent experiments (b), and n=3 independent experiments (c); mean ± SEM. Source data are provided as a Source Data file.

Supplementary Fig. 5

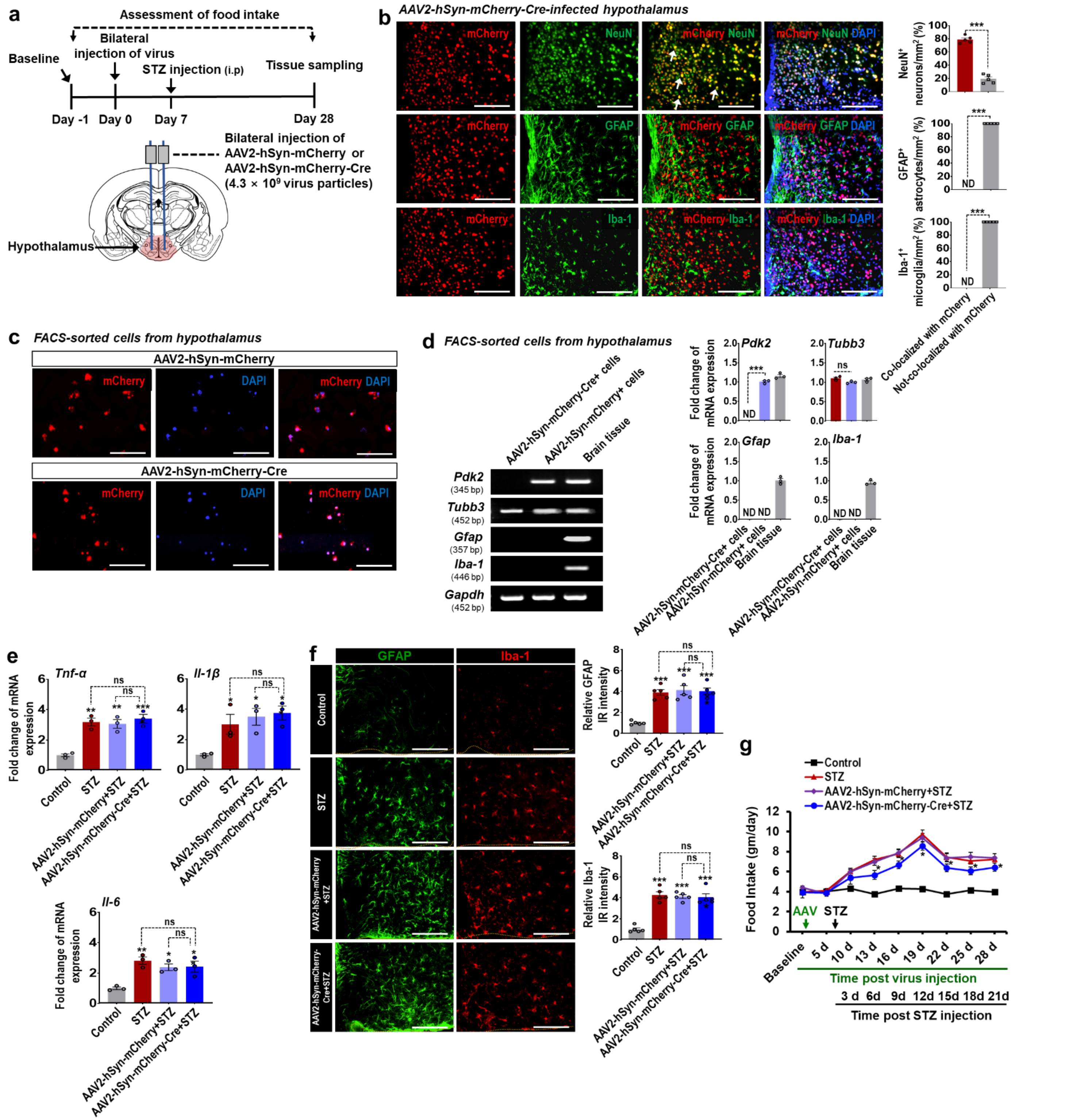

Supplementary Fig. 5 ***Effects of neuron-specific Cre-mediated Pdk2 gene ablation on diabetes-induced hypothalamic inflammation and changes in feeding behavior.*** To ablate *Pdk2* in hypothalamic neurons, we administered AAV2-hSyn-mCherry or AAV2-hSyn-mCherry-Cre particles (4.3×10^9 virus particles per side) stereotactically into the bilateral mediobasal hypothalamus of *Pdk2* floxed mice. The schematic diagram presents the experimental timeline and route of administration (a). NeuN, GFAP, and Iba-1 immunofluorescence staining of

brain tissues isolated from virus-injected *Pdk2* floxed mice confirmed an efficient virus infection specific for neurons after 4 w of virus administration (b). Arrows indicate mCherry co-localized cells. Quantification of the percentage of mCherry-positive or -negative NeuN-expressing neurons (***p= 2.6E-7, co-localized with mCherry), GFAP-expressing astrocytes (***p= 1.7E-15, not co-localized with mCherry), or Iba-1-expressing microglia (***p= 1.1E-15, not co-localized with mCherry) per mm^2 is shown with the adjacent images. FACS-sorted mCherry-positive hypothalamic cells were confirmed under fluorescent microscopy and used for RT-PCR analysis (c). The expression of *Pdk2* (***p= 8.3E-7, AAV2-hSyn-mCherry-Cre^+ cells), *Gfap*, and *Iba-1* mRNA in FACS-sorted mCherry-positive cells confirmed successful *Pdk2* recombination in hypothalamic neurons *in vivo* (d). mRNA expression profiles are displayed as the fold increase of gene expression normalized to *Gapdh*. The fold change was made for *Pdk2* and *Tubb3* against AAV2-hSyn-mCherry-infected cells as control and *Gfap* and *Iba-1* against brain tissue as control. The relative expression of *Tnf-α* (p= 0.7454, AAV2-hSyn-mCherry-Cre+STZ), *IL-1β* (p= 0.983, AAV2-hSyn-mCherry-Cre+STZ), and *IL-6* (p= 0.9995, AAV2-hSyn-mCherry-Cre+STZ) mRNA in the hypothalamic tissues isolated from AAV2-hSyn-mCherry-Cre or AAV2-hSyn-mCherry and STZ-injected mice was evaluated by real-time RT-PCR (e). GFAP (p= 0.9957, AAV2-hSyn-mCherry-Cre+STZ) and Iba-1 (p= 0.9977, AAV2-hSyn-mCherry-Cre+STZ) immunostaining analysis of hypothalamic tissues isolated from virus-injected mice after 3 w of STZ injection (f). All microscope data were accumulated using five randomly selected fields captured at the same magnification. Scale bars indicate 200 µm. Food intake (*p= 0.0388, AAV2-hSyn-mCherry-Cre+STZ at 28 d) was assessed following AAV2-hSyn-mCherry-Cre or AAV2-hSyn-mCherry and STZ administration (g). Arrows indicate the time points of adeno-associated virus (AAV) construct and STZ administration. *$p < 0.05$, **$p < 0.01$, or ***$p < 0.001$ versus the mCherry+ (b) or vehicle-treated control animals (e and f) or STZ/ AAV2-hSyn-mCherry+STZ-treated animals (g). One-way ANOVA (d-f), two-way ANOVA (g) with Tukey's post-hoc test, two-tailed Student's *t*-test (b), n=5 (b, f), n = 3 (c-e), and n = 6 (g); mean ± SEM. Value of 'n' indicates the number of animal. Source data are provided as a Source Data file. IR, immunoreactivity; d, day(s); ND, not detected; ns, not significant.

Supplementary Fig. 6

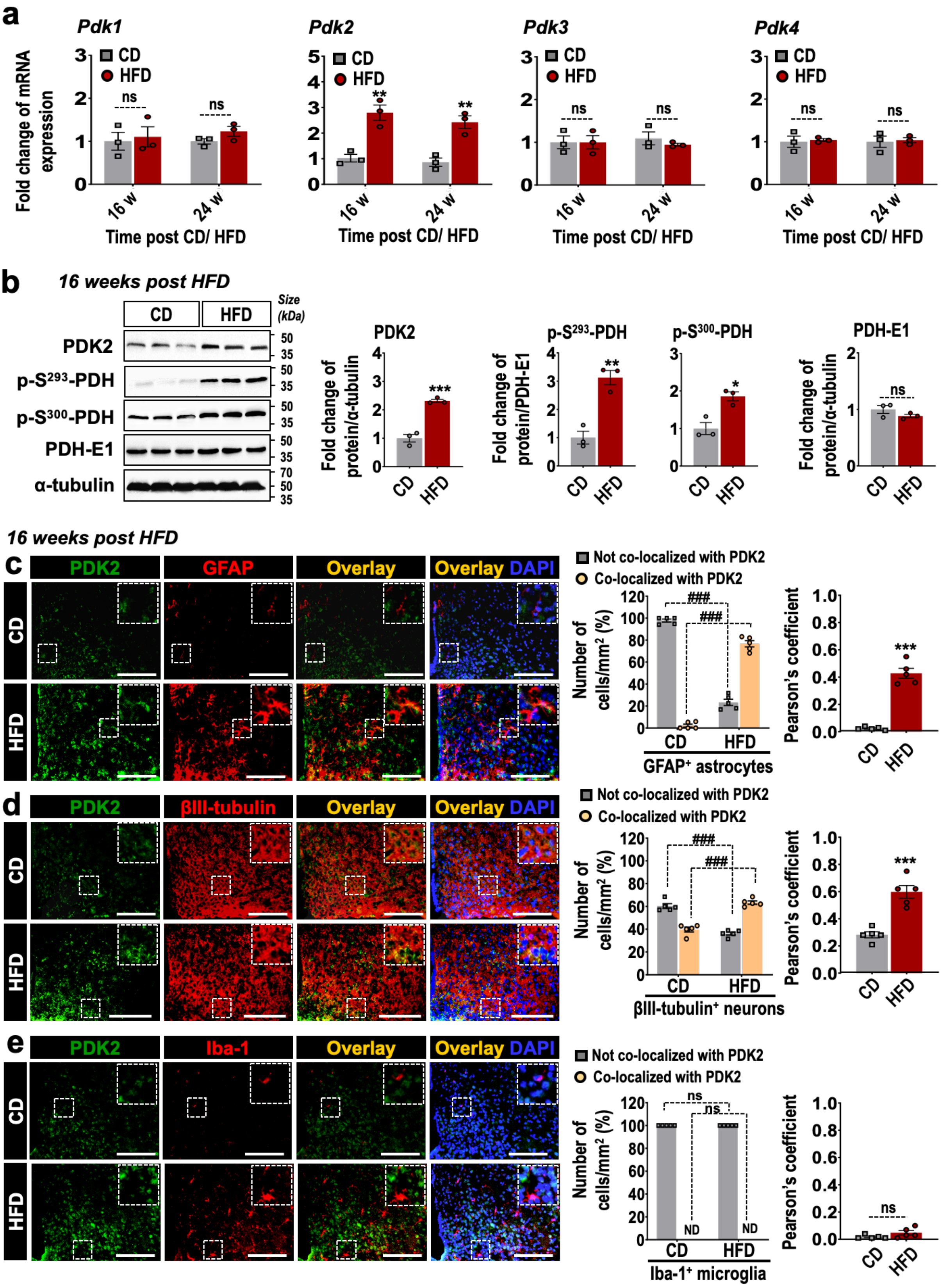

Supplementary Fig. 6 ***Expression of PDK and phosphorylated-PDH in the mouse hypothalamus following high-fat diet feeding.*** The expression of *Pdk isoforms (Pdk1-4)* mRNA (**p= 0.0061, HFD at 16 w; and **p= 0.0063, HFD at 24 w for *Pdk2*) in the hypothalamus at 16 and 24 w following HFD feeding (a) was assessed by real-time

RT-PCR. Results for mRNA expression are displayed as the fold increase of gene expression normalized to *Gapdh*. PDK2 (***p= 0.0008, HFD), phosphorylated-PDH (p-S^{293}-PDH: **p= 0.0034, HFD; and p-S^{300}-PDH: *p= 0.0127, HFD), and PDH-E1 protein levels in the hypothalamus at 16 w post-HFD feeding (b) were assessed by Western blot analysis. Quantification for p-PDH was based on normalization against PDH-E1, and PDK2 and PDH-E1 were normalized against α-tubulin. Immunofluorescence analyses show the expression of PDK2 in GFAP-positive astrocytes (c), βIII-tubulin-positive neurons (d), and Iba-1-positive microglia (e) in the hypothalamus at 16 w post-HFD feeding. Inserts show representative double-labeled cells. Quantification of the percentage of PDK2-positive or -negative astrocytes (###p= 5.5E-13, not co-localized with PDK2; and ###p= 5.5E-13, co-localized with PDK2), neurons (###p= 3.6E-7, not co-localized with PDK2; and ###p= 3.8E-7, co-localized with PDK2), or microglia. Pearson's correlation coefficient for co-localization per mm^2 (***p= 4.2E-6, HFD for panel c; and ***p= 0.0003, HFD for panel d) is shown in the adjacent graphs. Results were obtained from three different animals for each condition. Microscope data were gathered using five randomly selected fields captured at same magnification. Scale bar indicates 200 μm (c-d) and 100 μm (e). *p < 0.05, **p < 0.01, or ***p < 0.001 versus the vehicle-treated control animals. Two-way ANOVA with Tukey's post-hoc test (a, c-e; left); two-tailed Student's *t*-test (b, c-e; right), and n = 3 (a-b), and n=5 (c-e); mean ± SEM. Value of 'n' indicates the number of animal. Source data are provided as a Source Data file. w, week(s); CD, control diet; HFD, high-fat diet; ns, not significant; ND, not detected.

Supplementary Fig. 7

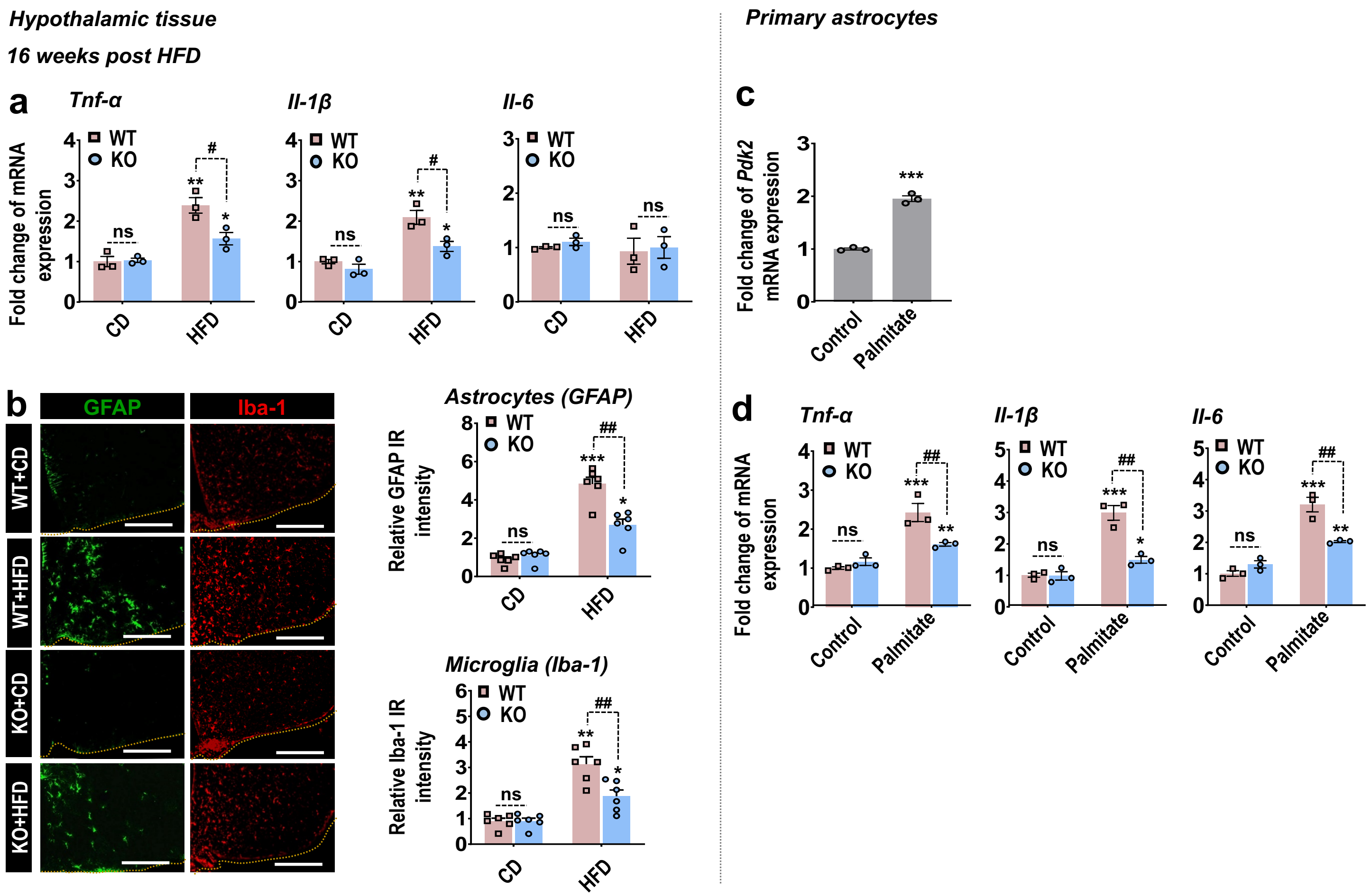

Supplementary Fig. 7 ***Pdk2 deficiency reduces high-fat diet/palmitate-induced inflammation in the mouse hypothalamus or cultured astrocytes.*** The relative expression of *Tnf-α* (#p= 0.0138, KO+HFD), *Il-1β* (#p= 0.0155, KO+HFD), and *Il-6* (#p= 0.9895, KO+HFD) mRNA in the hypothalamus after 16 w of HFD feeding (a) was evaluated by real-time RT-PCR. GFAP (##p= 0.0010, KO+HFD) and Iba-1 (##p= 0.0015, KO+HFD) immunofluorescence staining revealed increased immunoreactivity in the hypothalamus of HFD-fed WT mice, but *Pdk2* deficiency significantly attenuated the increase in immunoreactivity at 16 w post-HFD feeding (b). Microscope data were gathered using five to six randomly selected fields captured at the same magnification. Scale bar indicates 200 µm. Cultured astrocytes isolated from WT and *Pdk2*-deficient mice were treated with palmitate (200 µM) for 48 hr. The expression of (c) *Pdk2* (***p= 7.4e-5, palmitate), (d) *Tnf-α* (##p= 0.0012, KO+palmitate), *Il-1β* (##p= 0.0050, KO+palmitate), and *Il-6* (##p= 0.0014, KO+palmitate) mRNA was assessed by real-time RT-PCR. Results for mRNA expression are displayed as the fold increase of gene expression normalized against *Gapdh*. *p < 0.05, **p < 0.01, or ***p < 0.001 versus vehicle-treated control animals or non-treated cells; #p < 0.05 or ##p < 0.01 versus indicated groups. Two-way ANOVA with Tukey's post-hoc test (a, b, d), two-tailed Student's *t*-test (c), n = 3 independent animals (a) or sister wells (c-d), and n=6 independent animals (b); mean ± SEM. Source data are provided as a Source Data file. WT, wild-type; KO, knock out; CD, control diet; HFD, high-fat diet; IR, immunoreactivity; ns, not significant.

Supplementary Fig. 8

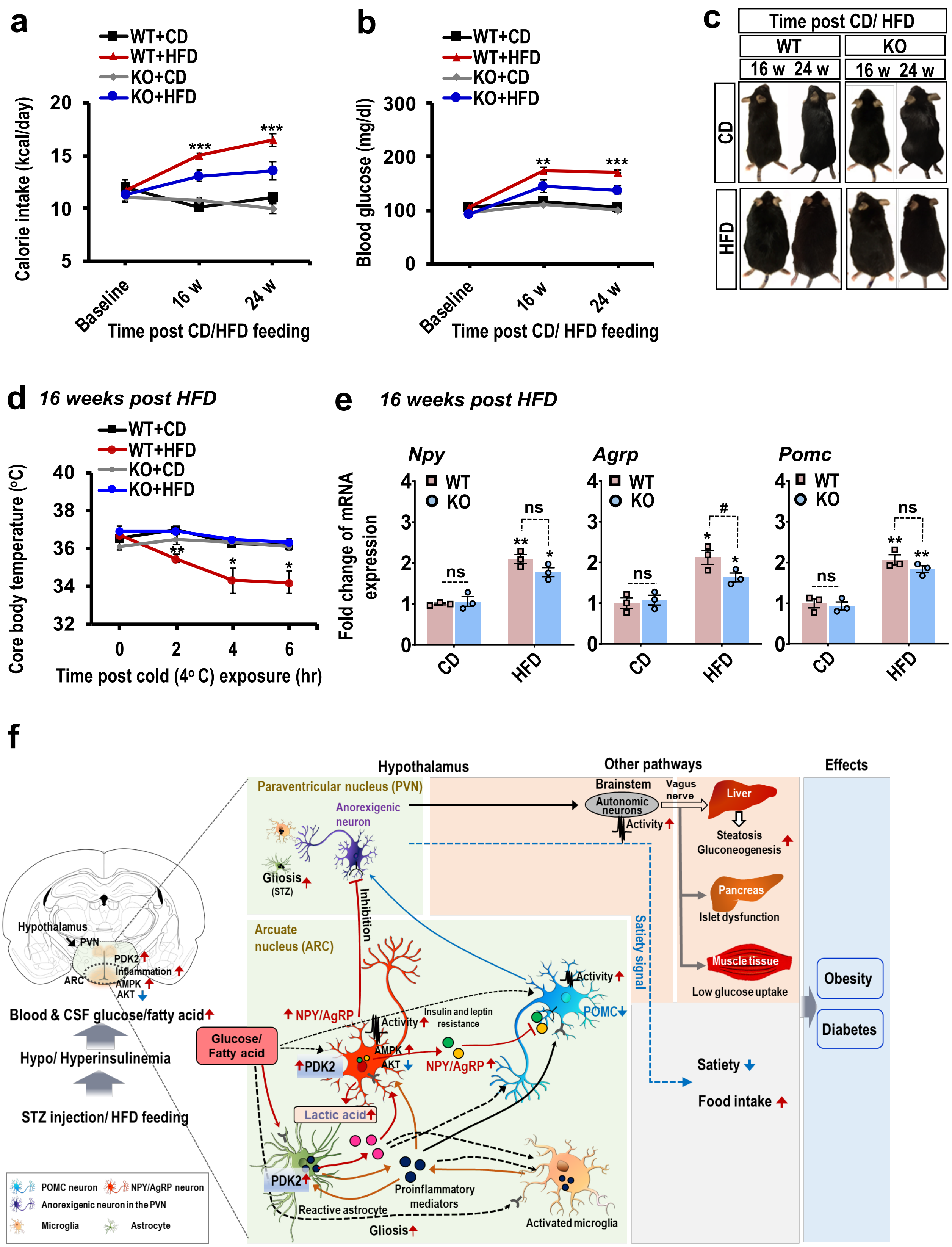

Supplementary Fig. 8 ***Pdk2 deficiency attenuates high-fat diet-induced increased calorie intake, hyperglycemia, body size, thermogenesis, and expression of orexigenic neuropeptides in mice.*** (a) Caloric intake (***p= 0.0002, KO+HFD at 16 w; and ***p= 0.0005, KO+HFD at 24 w), (b) fasted blood (**p= 0.0013, KO+HFD at 16 w; and

***p= 0.0006, HFD at 24 w), and (c) body size in mice were assessed following HFD feeding at indicated time points. (d) Core body temperature was assessed at 16 w post-HFD feeding (**p= 0.0020, KO+HFD at 2 hr; *p= 0.0102, KO+HFD at 4 hr; and *p= 0.0164, KO+HFD at 6 hr). The relative expression of *Npy* (p= 0.1762, KO+HFD), *Agrp* (#p= 0.0312, KO+HFD)*,* and *Pomc* (p= 0.4421, KO+HFD) mRNA in the hypothalamus after 16 w of HFD feeding (e) was evaluated by real-time RT-PCR. Results for mRNA expression are displayed as the fold increase of gene expression normalized against *Gapdh*. A schematic proposing an outline of the implications of astrocyte PDK2 in the hypothalamus following diabetes and obesity (f). *p < 0.05, **p < 0.01, or ***p < 0.001 versus the HFD-fed KO animals (a, b, d), or CD-fed WT/KO control animals (e). Two-way ANOVA with Tukey's post-hoc test, n = 5 (a, b, d), and n = 3 (e); mean ± SEM. Value of 'n' indicates the number of animals. Source data are provided as a Source Data file. w, week(s); d, day(s); WT, wild-type; KO, knock out; CD, control diet; HFD, high-fat diet; ARC, arcuate nucleus; PVN, paraventricular nucleus; CSF, cerebrospinal fluid; ns, not significant.
