# Supplementary material for: Astrocytic pyruvate dehydrogenase kinase-2 is involved in hypothalamic inflammation in mouse models of diabetes
Source: Nat Commun. 2020 Nov 20;11:5906. doi: 10.1038/s41467-020-19576-1 (PMC7680139; doi:10.1038/s41467-020-19576-1)
Supplement: Supplementary file 2 — Reporting Summary [file 41467_2020_19576_MOESM2_ESM.pdf]

nature research

Corresponding author(s): Kyoungho Suk

Last updated by author(s): 10/12/2020

Reporting Summary

Nature Research wishes to improve the reproducibility of the work that we publish. This form provides structure for consistency and transparency in reporting. For further information on Nature Research policies, see Authors & Referees and the Editorial Policy Checklist .

Statistics

For all statistical analyses, confirm that the following items are present in the figure legend, table legend, main text, or Methods section.

n/a	Confirmed	
☐	☒	The exact sample size (*n*) for each experimental group/condition, given as a discrete number and unit of measurement
☐	☒	A statement on whether measurements were taken from distinct samples or whether the same sample was measured repeatedly
☐	☒	The statistical test(s) used AND whether they are one- or two-sided<br>*Only common tests should be described solely by name; describe more complex techniques in the Methods section.*
☐	☒	A description of all covariates tested
☐	☒	A description of any assumptions or corrections, such as tests of normality and adjustment for multiple comparisons
☐	☒	A full description of the statistical parameters including central tendency (e.g. means) or other basic estimates (e.g. regression coefficient) AND variation (e.g. standard deviation) or associated estimates of uncertainty (e.g. confidence intervals)
☐	☒	For null hypothesis testing, the test statistic (e.g. *F*, *t*, *r*) with confidence intervals, effect sizes, degrees of freedom and *P* value noted<br>*Give P values as exact values whenever suitable.*
☒	☐	For Bayesian analysis, information on the choice of priors and Markov chain Monte Carlo settings
☒	☐	For hierarchical and complex designs, identification of the appropriate level for tests and full reporting of outcomes
☐	☒	Estimates of effect sizes (e.g. Cohen's *d*, Pearson's *r*), indicating how they were calculated

Our web collection on statistics for biologists contains articles on many of the points above.

Software and code

Policy information about availability of computer code

Data collection	Microslicer (VT1000S; Leica, Nussloch, Germany) for tissue slice; Leica Microsystems (DM2500) for fluorescence microscopy and data analysis; High-performance Liquid Chromatography (Shimadzu, LC-20AT) for lactate assay; Seahorse XF24 Analyzer(Seahorse Bioscience Inc.) for ECAR and OCR assay; Nikon (SMZ-1) and Axopatch amplifier (Molecular Devices, 200B) for electrophysiology; Automated high-speed cell sorter (Beckman Coulter, MoFlo XDP) for the validation of conditional knockout mice.
Data analysis	Fiji (ImageJ, version 1.47) for IF image analysis and quantifying of western blot bands; GraphPad Prism (version 8) for graphs and statistical analysis; GPower (version 3.1) for determination of sample size; Wave Software (version 2.6) for the analysis of seahorse data; pCLAMP version 10.6 (Molecular Devices) for storing the the results of membrane currents in a computer database; SPSS program version 26.0K (SPSS Inc., Chicago, IL) for the Kolmogorov-Smirnov (K-S) test; Summit software (version 5.2) for FACS analysis.

For manuscripts utilizing custom algorithms or software that are central to the research but not yet described in published literature, software must be made available to editors/reviewers. We strongly encourage code deposition in a community repository (e.g. GitHub). See the Nature Research guidelines for submitting code & software for further information.

Data

Policy information about availability of data

All manuscripts must include a data availability statement. This statement should provide the following information, where applicable:

- Accession codes, unique identifiers, or web links for publicly available datasets
- A list of figures that have associated raw data
- A description of any restrictions on data availability

The authors declare that all data supporting the findings of this study are available within the paper and its supplementary information files. All source data are available in the Source Data file, which contains raw data including Figs. 1–8 and Supplementary Figs. 1–8, brief description of all figures, sample size, and detailed statistical analyses.

Field-specific reporting

Please select the one below that is the best fit for your research. If you are not sure, read the appropriate sections before making your selection.

- [x] Life sciences
- [ ] Behavioural & social sciences
- [ ] Ecological, evolutionary & environmental sciences

For a reference copy of the document with all sections, see nature.com/documents/nr-reporting-summary-flat.pdf

Life sciences study design

All studies must disclose on these points even when the disclosure is negative.

Sample size	The sample size for each experiment was determined by power analysis. The statistical power was calculated before data collection based on information from previous studies to decide the sample size needed and then adjusted in cases when the result turned out to be non-significant. The power of the study was considered to be minimum 0.8 (PMID: 12954688 and 17695343).
Data exclusions	No data excluded from the study.
Replication	Experiments were repeated at least two to three independent experiments with similar results. The inter-assay coefficient of variation was <20%, which is considered to indicate confidence in the results.
Randomization	All samples were randomly assigned to each experimental group.
Blinding	Analyses were performed blinded to animal genotype and treatment. An experimenter blinded to animal genotype carried out all behavioral tests.

Reporting for specific materials, systems and methods

We require information from authors about some types of materials, experimental systems and methods used in many studies. Here, indicate whether each material, system or method listed is relevant to your study. If you are not sure if a list item applies to your research, read the appropriate section before selecting a response.

Materials & experimental systems

n/a	Involved in the study	
☐	☒	Antibodies
☐	☒	Eukaryotic cell lines
☒	☐	Palaeontology
☐	☒	Animals and other organisms
☒	☐	Human research participants
☒	☐	Clinical data

Methods

n/a	Involved in the study	
☒	☐	ChIP-seq
☐	☒	Flow cytometry
☒	☐	MRI-based neuroimaging

Antibodies

Antibodies used	Rabbit polyclonal PDK1 (Enzo Life Sciences, Cat# ADI-KAP-PK112-D, RRID: AB_2039453); Rabbit polyclonal PDK2 (Abgent, Cat# AP7039b, RRID: AB_2161467); Rabbit polyclonal PDK3 (Abnova, Cat# PAB4563); Rabbit polyclonal PDK4 (Atlas Antibodies, Cat# HPA056731, RRID: AB_2683218); Rabbit polyclonal phospho-Ser293-PDH-E1α (Calbiochem, Cat# AP1062, RRID: AB_10616069); Rabbit polyclonal phospho-Ser300-PDH-E1α (Calbiochem, Cat# AP1064); Rabbit monoclonal PDH-E1 (Cell Signaling Technology, Cat# 3205, RRID: AB_2162926); Rabbit monoclonal phospho-AMPKα (Thr 172) (Cell Signaling Technology, Cat# 2535, RRID: AB_331250); Rabbit monoclonal AMPKα (Cell Signaling Technology, Cat# 5832, RRID: AB_ 10624867); Rabbit polyclonal phospho-AKT (Cell Signaling Technology, Cat# 9271, RRID: AB_329825); Rabbit polyclonal AKT Cell Signaling Technology, Cat# 9272, RRID: AB_329827); Goat polyclonal Iba-1 (Novus Biologicals, Cat# NB100-1028, RRID: AB_2224398); Rabbit polyclonal Iba-1 (Wako Pure Chemical Corporation, Cat# 019-19741, RRID:AB_839504); Mouse polyclonal GFAP (Novus Biologicals, Cat# NB1-05197, RRID: AB_1555288); Rabbit polyclonal GFAP (DAKO, Cat# Z0334, RRID:AB_10013382); Mouse βIII-tubulin (Santa Cruz Biotechnology Inc., Cat# sc80005); NeuN (rabbit, 1:500, Merk Millipore, Cat# ABN78); Rabbit polyclonal AGRP (Phoenix Pharmaceuticals Inc., Cat# H-003-57, RRID:AB_2313909); Rabbit polyclonal POMC (Phoenix Pharmaceuticals Inc., Cat# H-029-30, RRID:AB_2307442); NF-kB p65 (Santa Cruz Biotechnology Inc., Cat# sc-8008, RRID:AB_628017); Mouse monoclonal α-tubulin (Cell Signaling Technology, Cat# T5168, RRID: AB_477579); Horseradish peroxidase-conjugated secondary antibody anti-rabbit (Cell Signaling Technology, Cat# 7074S, RRID: AB_2099233); Horseradish peroxidase-conjugated secondary antibody anti-mouse (Cell Signaling Technology, Cat# 7076S, RRID: AB_330924); Anti-Rabbit IgG, Biotinylated (Vector Laboratories, Cat# BA-1000; RRID: AB_2313606); FITC (code: 711-096-152, RRID: AB_2340597 or code: 715-095-151, RRID: AB_2335588) - or Cy3 (code: 705-165-147, AB_2307351 or code: 715-165-151, RRID: AB_2315777or code: 711-165-152, RRID: AB_2307443) or Cy5 (code: 711-175-152, RRID: AB_2340607)-conjugated secondary antibodies (Jackson ImmunoResearch).

Validation

Detail validation statement for each primary or secondary antibody is provided on the manufacturer's website.

Briefly,
Rabbit polyclonal PDK1 (Enzo Life Sciences, Cat# ADI-KAP-PK112-D): Species reactivity: Human, Mouse, Rat,Dog, Monkey, Porcine, Rabbit; Applications: IHC (PS), WB; citation: PMID: 31578313.
Rabbit polyclonal PDK2 (Abgent, Cat# AP7039b): Species reactivity: Human, Mouse, Rat; Applications: IHC-P, WB, E; citation: PMID: 22910903.
Rabbit polyclonal PDK3 (Abnova, Cat# PAB4563): Species reactivity: Human, Mouse; Applications: WB, E; citation: PMID: 18541534.
Rabbit polyclonal PDK4 (Atlas Antibodies, Cat# HPA056731): Species reactivity: Human, Mouse; Applications: WB, IHC; citation: PMID: 26769971.
Rabbit polyclonal phospho-Ser293-PDH-E1α (Calbiochem, Cat# AP1062) Species reactivity: Human, Mouse, Primate, Rat; Applications: WB, IP, IHC; citation: PMID: 26769971.
Rabbit polyclonal phospho-Ser300-PDH-E1α (Calbiochem, Cat# AP1064) Species reactivity: Human, Mouse; Applications: WB, IHC; citation: PMID: 26769971.
Rabbit monoclonal PDH-E1 (Cell Signaling Technology, Cat# 3205) Species reactivity: Human, Mouse Rat, Monkey; Applications: WB, IHC; citation: PMID: 26769971.
Rabbit monoclonal phospho-AMPKα (Thr 172) (Cell Signaling Technology, Cat# 2535): Species reactivity: Human, Mouse, Rat, Monkey etc.; Applications: WB, IP, IHC; citation: PMID: 32439975.
Rabbit monoclonal AMPKα (Cell Signaling Technology, Cat# 5832): Species reactivity: Human, Mouse, Rat, Monkey etc.; Applications: WB, IP; citation: PMID: 30909789.
Rabbit polyclonal phospho-AKT (Cell Signaling Technology, Cat# 9271) Species reactivity: Human, Mouse, Rat etc.; Applications: WB, IP, IHC; citation: PMID: 32140744.
Rabbit polyclonal AKT (Cell Signaling Technology, Cat# 9272) Species reactivity: Human, Mouse, Rat, Monkey etc.; Applications: WB, IP, IHC etc.; citation: PMID: 32312868.
Goat polyclonal Iba-1 (Novus Biologicals, Cat# NB100-1028): Species reactivity: Human, Mouse, Rat etc.; Applications: WB, ICC/IF, IHC, IHC-Fr, IHC-P, PEP-ELISA, Dual ISH-IHC, IHC-FrFl, IHC-WhMt, KO; citation: PMID: 32220118.
Rabbit polyclonal Iba-1 (Wako Pure Chemical Corporation, Cat# 019-19741): Species reactivity: Human, Mouse, Rat; Applications: IHC, ICC; citation: PMID: 28066237.
Mouse polyclonal GFAP (Novus Biologicals, Cat# NB1-05197) Species reactivity: Human, Mouse, Rat etc.; Applications: WB, Simple Western, ICC/IF, IHC, IHC-Fr, IHC-P, IHC-FrFl; citation: PMID: 25421913.
Rabbit polyclonal GFAP (DAKO, Cat# Z0334) Species reactivity: Human, Mouse, Rat etc; Applications: WB, IHC, ICC; citation: PMID: 28186121.
Mouse βIII-tubulin (Santa Cruz Biotechnology Inc., Cat# sc80005) Species reactivity: Mouse; Applications: WB, IHC, IF, IP; citation: PMID: 26490872.
NeuN (Merk Millipore, Cat# ABN78) Species reactivity: Mouse, Rat, Human; Applications: WB, IHC, ICC; citation: PMID: 26358247.
Rabbit polyclonal AGRP (Phoenix Pharmaceuticals Inc., Cat# H-003-57) Species reactivity: Mouse, Human; Applications: IF, IHC; citation: PMID: 17671657.
Rabbit polyclonal POMC (Phoenix Pharmaceuticals Inc., Cat# H-029-30) Species reactivity: Mouse; Applications: IHC, IF; citation: PMID: 23426689.
NF-kB p65 (Santa Cruz Biotechnology Inc., Cat# sc-8008) Species reactivity: Mouse; Applications: WB, IHC, IF; citation: PMID: 30326222.
Mouse monoclonal α-tubulin (Cell Signaling Technology, Cat# T5168) Species reactivity: Human, Mouse, Rat, Monkey etc.; Applications: WB, IHC, IF; citation: PMID: 26490872.
Horseradish peroxidase-conjugated secondary antibody anti-rabbit (Cell Signaling Technology, Cat# 7074S) Species reactivity: Mouse; Applications: WB; citation: PMID: 26490872.
Horseradish peroxidase-conjugated secondary antibody anti-mouse (Cell Signaling Technology, Cat# 7076S): Species reactivity: Mouse; Applications: WB; citation: PMID: 26490872.
Anti-Rabbit IgG, Biotinylated (Vector Laboratories, Cat# BA-1000): PMID: 32867800.
FITC (code: 711-096-152, or code: 715-095-151) - or Cy3 (code: 705-165-147 or code: 715-165-151, or code: 711-165-152) or Cy5 (code: 711-175-152)-conjugated secondary antibodies (Jackson ImmunoResearch): PMID: 26490872, 26769971, 32242017.

Eukaryotic cell lines

Policy information about cell lines

Cell line source(s)

The embryonic mouse hypothalamic-N41cells (mHypoE-N41) expressing Npy/AgRP and embryonic mouse hypothalamic-N43/5 (mHypoE-N43/5) cells expressing POMC (Cellutions Biosystems Inc., kindly provided by Professor Eun-Kyoung Kim at Daegu Gyeongbuk Institute of Science & Technology, Republic of Korea).

Authentication

The cell lines used in this study were authenticated by CEDARLANE CELLutions BIOSYSTEM INC. (Ontario, Canada).
The embryonic mouse hypothalamic-N41cells (mHypoE-N41): Code-CLU120.
The embryonic mouse hypothalamic-N43/5 (mHypoE-N43/5): Code-CLU127.
References:
https://www.cedarlanelabs.com/products/listing/hypothalamic?lob=Cellutions
PMID: 27533078.

Mycoplasma contamination	All the cells had been tested and confirmed negative for mycoplasma contamination.
Commonly misidentified lines (See ICLAC register)	No commonly misidentified cell lines were used.

Animals and other organisms

Policy information about studies involving animals; ARRIVE guidelines recommended for reporting animal research

Laboratory animals	Male mice aged 8–10 weeks were used in this study. Age-matched WT mice were produced from the C57BL/6J mice (The Jackson Laboratory, Bar Harbor, ME), which were used to stabilize the genetic backgrounds of the Pdk2 KO. The Pdk2 floxed mice were kindly provided by Professor In-Kyu Lee at Kyungpook National University (Daegu, Republic of Korea).
Wild animals	No wild animals were used in this study.
Field-collected samples	No field-collected samples were used in this study.
Ethics oversight	All experiments were conducted in accordance with approved animal protocols and guidelines established by the Animal Care Committee of Kyungpook National University (Approval No., KNU-2012-73/66).

Note that full information on the approval of the study protocol must also be provided in the manuscript.

Flow Cytometry

Plots

Confirm that:

- [x] The axis labels state the marker and fluorochrome used (e.g. CD4-FITC).
- [x] The axis scales are clearly visible. Include numbers along axes only for bottom left plot of group (a 'group' is an analysis of identical markers).
- [x] All plots are contour plots with outliers or pseudocolor plots.
- [x] A numerical value for number of cells or percentage (with statistics) is provided.

Methodology

Sample preparation	Hypothalamic tissues were isolated from AAV5-GFAP-eYFP- or AAV5-GFAP-mCherry-Cre or AAV2-hSyn-mCherry or AAV2-hSyn-mCherry-Cre-injected mice, and then minced and further dissociated. Briefly, tissues were incubated with a solution of 1 X Accutase (ThermoFisher) supplemented with 80 U/ml DNase I (Sigma) at 37°C for 20 min, followed by gentle trituration in Hybernate A media (Invitrogen) along with 1% FBS. The cell suspension was prepared by passing it through a 70 µm filter, overlaying it on top of an isotonic Percoll gradient (top phase: 11%, 31 ml; bottom phase: 30%, 2 ml), and centrifuging it at 400X g for 5 min at 4°C. Dissociated mixed cells were retrieved using a pipette carefully from the interface between 11% and 13% phases and pelleted by centrifugation. The cell pellet was washed twice and resuspended in 500 µl 1X PBS + 3% FBS, and then subjected to FACS.
Instrument	Automated high-speed cell sorter (Beckman Coulter, MoFlo XDP)
Software	Summit (version 5.2)
Cell population abundance	Two hundred thousands mCherry- or eYFP-positive cells were sorted from tissue samples and used for total RNA extraction and gene expression analysis.
Gating strategy	Negative control cells and mCherry- or eYFP-positive cells were divided into R1 and R2 gates based on forward/side scatter followed by doublet exclusion. We confirmed the mCherry- or eYFP-positive cells under fluorescent microscope just after FACS sorting. The data are shown as Supplementary Fig. 4B and 5C

- [x] Tick this box to confirm that a figure exemplifying the gating strategy is provided in the Supplementary Information.
